# Development of niosomal nanoparticles loaded with cisplatin and vorinostat combination for cancer therapy

**DOI:** 10.1371/journal.pone.0342344

**Published:** 2026-02-06

**Authors:** Hazar Ali, Zainab Lafi, Naeem Shalan

**Affiliations:** 1 Department of Pharmaceutics and Pharmaceutical Technology, Faculty of Pharmacy, Al-Ahliyya Amman University, Amman, Jordan; 2 Pharmacological and Diagnostic Research Center, Faculty of Pharmacy, Al-Ahliyya Amman University, Amman, Jordan; Dubai Pharmacy College, UNITED ARAB EMIRATES

## Abstract

Cisplatin (CIS) remains a cornerstone of chemotherapy but is limited by resistance and systemic toxicity. Combining DNA-damaging agents with epigenetic modulators such as vorinostat (VOR) offers a promising strategy to enhance efficacy. However, the co-delivery of these drugs is challenging due to their distinct physicochemical properties. The aim was to develop and characterize niosomal nanoparticles co-loaded with CIS and VOR (NIO-CIS-VOR) and to assess their physicochemical characteristics and in vitro anticancer activity. Niosomes were prepared using ethanol injection, with CIS entrapped in the aqueous core and VOR in the lipid bilayer. Characterization included particle size, polydispersity index (PDI), and zeta potential by DLS, morphology by TEM, and encapsulation confirmation by FTIR. Encapsulation efficiency (EE%) and drug release were determined by HPLC. Cytotoxicity, caspase-3/7 activation, colony formation, and wound healing assays were conducted in HT-29, A549, and PANC-1 cancer cell lines. Optimized NIO-CIS-VOR nanoparticles exhibited a mean diameter of 152.7 nm, PDI of 0.12, and zeta potential of –9.79 mV, with spherical morphology. Encapsulation efficiency of NIO-CIS-VOR reached 89.3% for CIS and 52.1% for VOR. The formulation showed sustained release over 72 h, with cumulative release of 62% (CIS) and 38% (VOR) at 6 h. Cytotoxicity assays demonstrated markedly reduced IC50 values for NIO-CIS-VOR compared with free drugs: 1.8 µM vs. 4.47 µM (CIS) and 3.4 µM (VOR) in HT-29; 0.95 µM vs. 3.8 µM and 3.1 µM in A549; and 2.37 µM vs. 13.9 µM and 3.66 µM in PANC-1. Enhanced apoptosis and reduced colony formation further confirmed superior anticancer activity.In Conclusion the Co-loaded niosomes achieved efficient co-delivery, sustained release, and synergistic anticancer effects, highlighting NIO-CIS-VOR as a promising nanocarrier for combination cancer therapy.

## 1. Introduction

**Fig 1:**
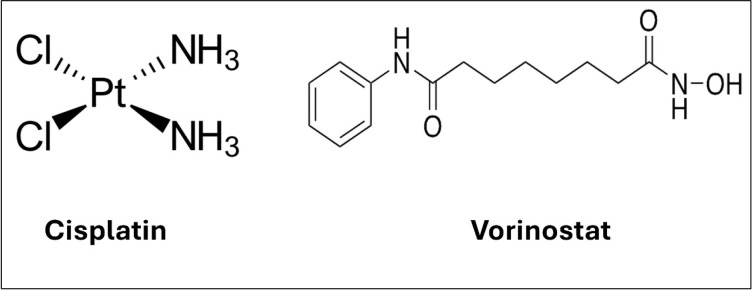
Chemical structures of CIS and VOR.

Cisplatin (CIS) remains one of the most widely used chemotherapeutic agents; however, its clinical success is hindered by resistance and dose-limiting toxicity [1]. Recent approaches suggest that combination therapy with histone deacetylase inhibitors (HDACIs), such as Vorinostat (VOR), may overcome these limitations by modulating epigenetic pathways and sensitizing cancer cells to chemotherapy [2,3]. Despite the therapeutic potential of CIS-VOR combination, their concurrent delivery remains a challenge due to differences in solubility and pharmacokinetics (Fig 1) [4]. Nanocarrier systems, particularly niosomes, non-ionic surfactant-based vesicles, offer a promising platform for simultaneous delivery of hydrophilic and lipophilic agents. Niosomes provide biocompatibility, enhanced stability, and the ability to encapsulate multiple drug types [5]. Niosomes are nanoscale spherical vesicles capable of loading a diverse range of drugs. Composed of amphiphilic molecules, they can encapsulate both hydrophilic and hydrophobic drugs [6,7]. These amphiphilic molecules form bilayer membranes, which allow the niosomes to exist either as unilamellar (with a single bilayer) or multilamellar (with multiple bilayers forming concentric spheres) depending on the synthesis method used [8]. To enhance the stability and other properties of the vesicles, non-ionic surfactants, cholesterol, or their derivatives are often incorporated during synthesis [8]. Various molecules, including amides, amino acids, alkyl ethers, alkyl esters, and fatty acids, along with surfactants such as alkyl esters (e.g., Tweens, Spans) and alkyl ethers (e.g., Brij), are used to create niosomes are able to trap hydrophilic drugs in their aqueous core and lipophilic drugs within their bilayer structure, making them versatile carriers for the delivery of drugs, hormones, and antigens [9].

Although niosomes share many similarities with liposomes, they differ in several key aspects. Both are spherical vesicles that can be unilamellar or multilamellar, formed by amphiphilic molecules, and their size typically in the same size ranges. Both are biocompatible and widely used in drug delivery systems [10,11].

This study focuses on the formulation, characterization, and anticancer evaluation of niosomal nanoparticles co-loaded with CIS and VOR. The hypothesis is that co-loaded niosomes can improve drug solubility, sustain release, and enhance therapeutic efficacy across multiple cancer cell lines.

## 2. Materials and methods

### 2.1 Materials

Cisplatin Powder (CAS No. 5663-27-1) and Vorinostat (N1-Hydroxy-NB-Phenyloctanediamide CAS No. 149647-78-9) powder were purchased from certified commercial suppliers (Sigma-Aldrich, St. Louis, MO, USA). Both compounds were of analytical grade and were used without further modification. Non-ionic surfactants (Tween 80), cholesterol, ethanol, and analytical-grade solvents were used for formulation. Cell culture media (DMEM), FBS, antibiotics, and other reagents were obtained from standard biological suppliers. The Cell Lines used in this study were obtained from ATCC, Lung Carcinoma, Non-small Cell Lung Cancer (A549 with ATCC No. CCL-185), Colorectal Adenocarcinoma (HT-29 with ATCC No.HTB-38) and Pancreatic Cancer, Ductal Adenocarcinoma (Panc-1, with ATCC No. CRL-1469)

### 2.2. Preparation of niosomal nanoparticles

**Fig 2:**
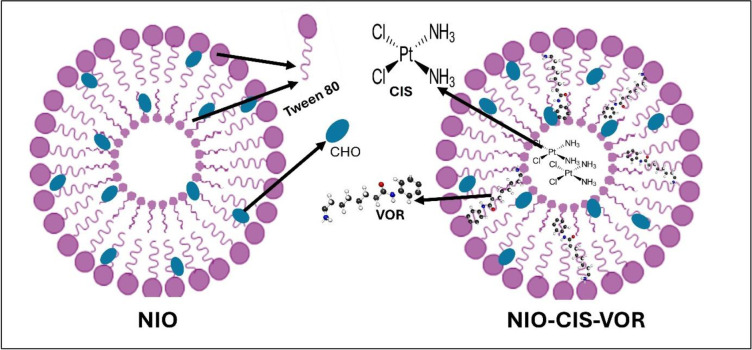
Schematic illustration of Niosomes and their components.

Niosomal nanoparticles were prepared using the ethanol injection method, a widely employed technique known for its simplicity and effectiveness in producing uniform vesicles. The formulation process began by preparing the organic phase of (surfactant: cholesterol, 1:3.25), which consisted of 25 mg (19.1 µmol ≈ 24 µL) of the non-ionic surfactant tween and 24 mg (62.1 µmol) of cholesterol, both dissolved in 1 mL of ethanol. This mixture was subjected to vigorous vortexing to ensure complete homogenization. Following this, the organic mixture was gently heated on a hotplate maintained at a temperature range of (50–70) °C, monitored using a calibrated laboratory thermometer. The heating step was essential to enhance the miscibility of the lipid components and promote uniform vesicle formation upon contact with the aqueous phase [12–15]. The preheated organic phase was then added dropwise into 3 mL of Phosphate-Buffered Saline (PBS) contained in a rounded-bottom flask. This was carried out under continuous magnetic stirring to facilitate the spontaneous formation of niosomal vesicles as the ethanol diffused into the aqueous medium. The dropwise addition allowed for controlled mixing and efficient self-assembly of the amphiphilic molecules into bilayer vesicular structures. The mixture was left to stir until the complete diffusion of Ethanol was achieved, and the dispersion became visually uniform, indicating successful formation of niosomal nanoparticles. For the preparation of drug-loaded formulations, same approach was followed. In the case of NIO-CIS, 0.5 mg of CIS (1.67 mM) was dissolved in 0.5 mL of normal saline to ensure complete solubilization. This solution was then added to the suspension of PBS and organic phase. On the other hand, for NIO-VOR, 0.5 mg of VOR (1.89 mM), being a lipophilic compound, was incorporated directly into the organic phase by dissolving it in the ethanol-tween-cholesterol mixture. This approach facilitated the localization of VOR within the lipid bilayer of the resulting niosomes. To develop a dual-loaded niosomal formulation (NIO-CIS-VOR), 0.5 mg of each drug, CIS and VOR, was used. The same respective procedures were applied;

VOR was introduced into the organic phase then injected into the PBS while the CIS solution was then added to the PBS-organic phase mixture. After completion of the injection, the resulting dispersion was stirred further to ensure uniformity and allow for full vesicle maturation (Fig 2).

### 2.3. Fourier-Transform Infrared (FTIR) spectroscopy

To investigate the potential interactions between the encapsulated drugs and the niosomal matrix, FTIR analysis was performed on the blank formulation (NIO) and the NIO-CIS-VOR. Prior to analysis, both formulations were mixed with an appropriate amount of sucrose to serve as a cryoprotectant and stabilizer during lyophilization. The samples were vortexed thoroughly to ensure uniform mixing and subsequently stored at −70 °C for at least 12 hours to allow for complete freezing. The frozen mixtures were then subjected to freeze-drying for 24 hours to obtain powdered samples suitable for IR Analysis. The freeze-dried powders were analyzed using FTIR spectroscopy to detect any characteristic shifts or changes in peak positions that could indicate chemical interactions between the niosomal components and the loaded drugs. The measurements were conducted at ambient room temperature. The resolution for these measurements was set at 8 cm^−1^ and 32 cm^−1^, scanning the range between (400–4000) cm^i1^. This range covers most of the infrared region and, thus, captures various functional groups that may be present in the samples. The resulting data were compared with the spectra of individual components, including blank niosomes, CIS, VOR, and Sucrose, to confirm successful encapsulation and compatibility of the drug within the vesicular matrix [16].

### 2.4. Transmission Electron Microscopy (TEM) analysis

Following the characterization of the niosomal NPs using Zetasizer, Transmission Electron Microscopy (TEM) was employed to further investigate the morphology and structural details of the nanoparticles. For TEM Analysis, a drop of the freshly prepared niosomal suspension was placed onto a carbon-coated copper grid using a micropipette. The sample was allowed to settle for 2–3 minutes to enable adequate adsorption of the vesicles onto the grid surface. Excess fluid was carefully removed using filter paper without disturbing the settled particles. The grid was then negatively stained by adding a drop of 2% (w/v) Phosphotungstic Acid (pH adjusted to 6.8) to enhance the contrast of the vesicle outlines. After staining, the grid was left to air-dry completely at room temperature in a dust-free environment. The dried samples were then examined using a TEM operating at an accelerating voltage of 80–120 kV. Images were captured at various magnifications to assess vesicle morphology and size consistency. The micrographs confirmed the spherical shape and smooth surface of the niosomal nanoparticles, as well as the absence of aggregation, indicating successful formulation and stability at the nanoscale. This analysis provided direct visual evidence supporting the formation of well-dispersed, spherical niosomal vesicles and complemented the findings from Size and Zeta Potential measurements [17].

### 2.5. HPLC method for quantification of CIS and VOR

To quantify the amount of CIS and VOR encapsulated within the niosomal NPs, a High-Performance Liquid Chromatography (HPLC) Method was developed and optimized. To establish this HPLC Method, a calibration curve was prepared using different concentrations of CIS and VOR. For both CIS and VOR, the concentration range was set between 0.015 and 1 mg/mL. Each concentration in the calibration curve was analyzed using HPLC. The resultant data, specifically the peak areas corresponding to each concentration, were then used to construct calibration curves, plotting concentration against peak area for both CIS and VOR. The linearity of these curves was critically assessed to confirm the reliability and accuracy of the HPLC method as per the International Council for Harmonisation (ICH) guidelines [18]. For the HPLC analysis, conditions were optimized using a C18 reverse phase column. The flow rate was consistently set at 0.7 mL/min, and the column temperature was maintained at 40°C. Each run involved a 10 µL sample injection and lasted for 6 minutes. For CIS, the mobile phase comprised a mixture of 80% methanol and 20% saline. On the other hand, for VOR detection, the mobile phase comprised of 80% methanol and 20% deionized water. The detection was carried out at a wavelength of 243 nm for VOR and at 204 nm for CIS. To quantify the Encapsulation Efficiency (EE%) within the niosomal formulations, a volume of 200 µL of the niosomal NPs suspension was mixed with 800 µL of methanol and subjected to constant sonication for 10 minutes at a temperature of 35°C ensuring the breakdown of the nanoformulations and complete release of the encapsulated drug(s). Following sonication, the mixture was subjected to high-speed centrifugation at 12,000 RPM to separate the released drug content from insoluble components. Finally, the supernatant was analysed using HPLC at a wavelength of 243 nm for VOR and at 204 nm for CIS.

The EE% was calculated according to the equations detailed below:


$$E\;E\%=\frac{weight\;of\;the\;entrapped\;drug\left(s\right)}{drug\;mass\;added\;to\;the\;formulation}\;\times \;100\%$$

### 2.6. Stability of Niosomal Formulations at 4 °C

To evaluate the physical stability of the prepared niosomal formulations over the time of experiment, a short-term stability study was conducted under refrigerated conditions (4 ± 1 °C) for a period of 60 days. Three formulations were assessed: NIO-CIS, NIO-VOR, and NIO-CIS-VOR. Freshly prepared formulations were stored in tightly sealed glass vials and kept in a refrigerator (4 °C) protected from light. At predefined time intervals (day 0, 1, 3, 7, 14, 21, 30, 45, and 60), aliquots were withdrawn and analyzed for size, PDI, and zeta potential. All measurements were carried out at 25 ± 1 °C after equilibrating the samples for 2 minutes. The stability of the formulations was assessed based on changes in particle size and zeta potential over time, with significant fluctuations suggesting potential aggregation, vesicle fusion, or surface charge destabilization.

### 2.7. *In vitro* drug release at physiological conditions

The *in vitro* drug release from the drug-loaded niosomal NPs was methodically examined using a Dialysis Method. To initiate the study, 0.25 mg of the freeze-dried drug-loaded niosomal NPs suspension was resuspended in 500 µL of PBS or saline and then placed inside a dialysis tubing cellulose membrane with a molecular weight cut-off (MWCO) of 100 kDa, corresponding to an estimated pore diameter of approximately 10 nm. the dialysis membrane was then immersed in 40 mL of PBS under constant stirring. Free drug(s) were used as a control. To monitor the drug release over time, 50 µL samples were withdrawn from the reservoir at predetermined time intervals (0, 30 minutes,1, 2, 3, 4, 5, 6, 24, 26, 28, 48 and 72 hours) and immediately replaced with an equal volume of fresh PBS to maintain sink conditions. The withdrawn samples were subsequently analysed using HPLC to quantify the cumulative released drug. The release percentage was calculated using the following formula:


$$Cumalative\;Release\;\%=\;\frac{Cumulative\;amount\;of\;drug\;released\;at\;time\;t}{Total\;drug\;amount)}\times \;100\%$$

### 2.8 Cytotoxicity study (MTT Assay)

A total number of 5x10^3^ cells in 100 μL of growth media were seeded in a 96-well plate and incubated overnight to allow cells adherence on the plate. Next day, cells were treated in triplicates with various concentrations (1.6–100 μM) of free CIS, free VOR, NIO-CIS, Nio-Vor, CIS-VOR, or the NIO-CIS-VOR formulation with an ultimate volume of 200 μL in each well. Also, appropriate control wells were included and consisted of untreated cells as a negative control. The cells were left in the incubator for a period of 72 hours. The cell viability was assessed using MTT Assay. At the 72 hours designated time point, the cells were treated with 0.05 mg/mL of MTT Reagent and left inside incubator for 4 hours in order to allow formation of formazan crystals. In the subsequent phase, the old media was discarded, and formazan crystals were dissolved in 150 μL of DMSO and left in the shaker in order to have all formazan crystals fully solubilize. Finally, the absorbance was read at 590 nm on BioTek Cytation 5 multi-mode plate reader. The absorbance at 630 nm was also measured and used as a background reference wavelength. The normalized Optical Density (OD) value was calculated as follows:

The Cell Viability was expressed as viability percentage relative to untreated control cells according to the following equation:


$$Viability\;\%=\;\frac{OD\;of\;treated\;cells}{OD\;of\;untreated\;control\;cells}\;\times \;100\%$$

### 2.9 Apoptotic Caspase Activity Assay (Caspase-3/7)

#### 2.9.1 Protein extraction and protein quantification.

1x10^6^ of HT-29, A549, and Panc-1 cells were seeded inside 6-well culture plates and incubated overnight to allow cell adhesion. Next day, cells were treated with free and niosomal formulations as follows: 2 μM of CIS, 1 μM of VOR, or equivalent concentrations of the mix for 72-hours. At the designated time-point, the cells were washed, trypsinized, and transferred into 15 ml falcon tubes along with the old media containing the dead cells. After that, cells were centrifuged at 500*g* for 5 minutes and the supernatant was discarded. The remaining cell pellet was lazed by applying a customized amount of ice-cold Radio-immunoprecipitation (RIPA) Buffer fortified with Protease Inhibitor Cocktail Tablet (Roche, UK). Finally, cell lysates were transferred into new Eppendorf Tubes and stored at −80 C for future use.

In order to gauge the protein concentration in disintegrated cells, Bicinchoninic Acid (BCA) Assay (Pierce, UK) was employed. 2 μL of the protein was added in triplicate into a clear 96-well plate along with serial dilutions of Bovine Serum Albumin (BSA) (Category NO: SV30160.03) with concentrations of (0.125–2 µg/ml). Following that, each well received 200 µL of BSA Reagent (Reagent A to B 50:1) and later the plate was incubated under the temperature of 37 ºC for 30 minutes. At the end, UV absorbance was measured at 570 nm employing a BioTek Cytation 5 multi-mode plate reader tool and the protein make up was calculated through comparison with BSA standard.

#### 2.9.2 Caspases-3/7 activity.

The cells were lysed, and the protein was extracted and quantified as previously discussed. The activity of Caspase-3/7 was measured using the Caspase-3/7 Calorimetric Assay Kit. Briefly, 50 µg of protein was transferred into a 96-well plate. On top of that, 50 µL of 2X reaction buffer (containing 100 mM of DDT) and 5 μL of the 4 mM DEVD-*p*-NA substrate (200 μM final concentration) were added. The mixture was mixed well and incubated at 37°C for 60 minutes. Eventually, the absorbance was read at OD of 400 nm on a BioTek Cytation 5 multi-mode plate reader [19].

### 2.10 Cell migration assay

The cell migration assay was conducted to evaluate the potential therapeutic effects of the niosomal formulations on cell migration process over a 48-hour period. Cells were seeded at a density of 2x10^6^ cells per well into a 12-well plate under standard conditions until they reached confluency. A scratch was then introduced across the cell monolayer using a sterile pipette tip to create a wound. The cells were then treated with free drugs, drug-loaded niosomes, or empty-niosomes.

After treatment, the cells were incubated for 48 hours, during which images of the wound area were captured at regular intervals of 24 hours using an Inverted Microscope. Finally, images of the scratches were captured before and during cell treatment using a Phase Contrast Microscope (model P. MICRO-001, Nikon) equipped with a 4 × magnification objective (Alquraishi et al., 2023). The Motic Images Plus version 2.0 software was employed to calculate the wound closure area (µm^2^). The wound closure was calculated as follows:

$\begin{array}{c} Wound\;closure(\%)\;=\;\frac{Area\;at\;time\;(0)-Area\;at\;time\;(t)}{Area\;at\;time\;(0)}\times \;100\% \end{array}$

### 3.18 Data analysis

The findings were expressed as the mean ± standard deviation of a minimum of three independent experiments. Significance was assessed using various statistical tests Paired t Test and one-way ANOVA. A value of <0.05 was deemed indicative of a statistically significant distinction. The analyses were conducted utilizing GraphPad Prism 9 (GraphPad Software Inc., USA). CompuSyn.exe (Version 1) and Microsoft Office Excel (Microsoft, USA). The Motic Images Plus version 2.0 software for migration assay calculation.

## 3. Results

### 3.1. High-Performance Liquid Chromatography (HPLC)

**Fig 3:**
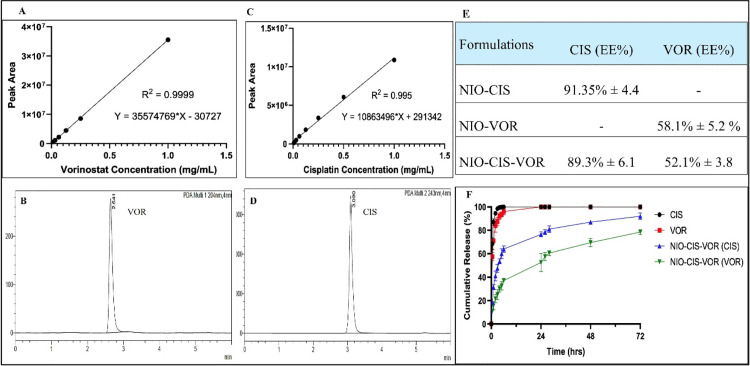
Characterization of CIS and VOR formulations. (A) Calibration curve of vorinostat showing linearity between concentration and peak area. (B) Representative HPLC chromatogram of vorinostat. (C) Calibration curve of cisplatin showing linearity between concentration and peak area. (D) Representative HPLC chromatogram of cisplatin. (E) Encapsulation efficiency (EE%) of CIS and VOR in single- and co-loaded niosomal formulations (NIO-CIS, NIO-VOR, NIO-CIS-VOR). (F) In vitro cumulative release profile of free drugs (CIS, VOR) and drug-loaded niosomal formulations (NIO-CIS-VOR)(Data are presented as mean ± SD (n = 3).

The linearity of the developed method was also validated by creating calibration curves using standard solutions across a concentration range of 0.015 and 1 mg/mL for both CIS and VOR. Linearity testing is essential to establish that the response of the detector (i.e., the peak area) is directly proportional to the concentration of the analyte within this range. The method demonstrated excellent linearity, with correlation coefficients (R²) of 0.995 for CIS and 0.9999 for VOR (Figs 3A,3B,3C, 3D). This high degree of linearity indicates that the method can reliably quantify varying concentrations of the analytes, which is critical for accurately determining the encapsulation efficiency and release profiles in subsequent experiments [18].

### 3.2. Evaluating the encapsulation efficiency of CIS and VOR

After developing and validating the method, the Encapsulation Efficiency (EE%) for CIS and VOR in the niosomal formulations was determined using the previously established calibration curves to determine the effectiveness of the nanoparticle formulations in incorporating these therapeutic agents. As depicted in (Fig 3E). The encapsulation efficiency (EE%) of cisplatin (CIS) in NIO-CIS was 91.35%, whereas a slightly lower value (89.3%) was observed in the co-loaded NIO-CIS-VOR formulation. For vorinostat (VOR), the EE% in NIO-VOR was 58.1%; however, upon co-encapsulation with CIS in NIO-CIS-VOR nanoparticles, the EE% of VOR decreased marginally to 52.1%.

### 3.3. In vitro drug release

The release profile of therapeutic agents from nanocarrier systems plays a pivotal role in determining their pharmacokinetics, therapeutic efficacy, and safety. In this study, the *in vitro* release behavior of CIS and VOR was systematically investigated in both their free forms and when co-encapsulated within niosomal vesicles (NIO-CIS-VOR) (Fig 3F).

The unencapsulated forms of CIS and VOR served as controls and exhibited a rapid release pattern consistent with typical burst kinetics. This behavior reflects the unrestricted diffusion of free drug molecules in the absence of a carrier system. CIS displayed a pronounced burst release, with approximately 65% of the drug released within the first 30 minutes. This value increased to nearly 98% within 3 hours, and complete release (~100%) was observed by 6 hours. The rapid release is primarily attributed to the high aqueous solubility of CIS and low molecular weight, facilitating rapid diffusion across the dialysis membrane. In contrast, free VOR exhibited a slightly slower yet still rapid release profile. Approximately 55% was released within 30 minutes, rising to about 85% at 3 hours, and reaching complete release by 24 hours. The marginal delay in release may be attributed to the amphiphilic nature of VOR and relatively lower solubility compared to CIS. The NIO-CIS-VOR formulation, wherein both CIS and VOR were co-encapsulated within niosomal vesicles, exhibited a distinctly controlled release behavior. This profile contrasts sharply with the free drug counterparts and highlights the capability of niosomal carriers to modulate drug release. At 0.5 hours, only 18% of CIS and 12% of VOR were released, indicating the initial protective effect of the niosomal bilayer, which limits the immediate diffusion of encapsulated agents. By 6 hours, cumulative release had progressed to 62% for CIS and 38% for VOR, signaling the onset of a sustained release phase. At 24 hours, the release reached 75% for CIS and 58% for VOR. By the end of the 72-hour study, cumulative release values approached 94% and 80% for CIS and VOR, respectively. This biphasic release behavior—characterized by an initial limited burst followed by a prolonged sustained phase—is typical of vesicular systems. It reflects the dual nature of drug localization: loosely bound or surface-associated molecules contribute to the initial release, while more deeply embedded or bilayer-integrated drug fractions are released gradually over time. The comparatively slower release of VOR may be attributed to its greater lipophilicity, which promotes its integration into the hydrophobic regions of the niosomal bilayer. These stronger drug–membrane interactions hinder its diffusion and prolong its release. Conversely, CIS, being hydrophilic, is likely distributed in the aqueous core or near the vesicle surface, allowing for faster release despite encapsulation. A clear distinction was observed between the release kinetics of the free drugs and the NIO-CIS-VOR formulation. While the free forms of CIS and VOR were rapidly and completely released within hours, their encapsulated counterparts exhibited a prolonged release over 72 hours. This confirms the capability of the niosomal carrier to modulate and extend the drug release profile. Notably, the co-encapsulation of CIS and VOR did not result in antagonistic release behavior. Instead, the dual-drug formulation supported a complementary and temporally coordinated release, which is particularly important in combination chemotherapy. Synchronized drug release can enhance synergistic interactions between agents and improve therapeutic outcomes.

### 3.4. Niosomal hydrodynamic diameter, zeta potential and stability

**Fig 4:**
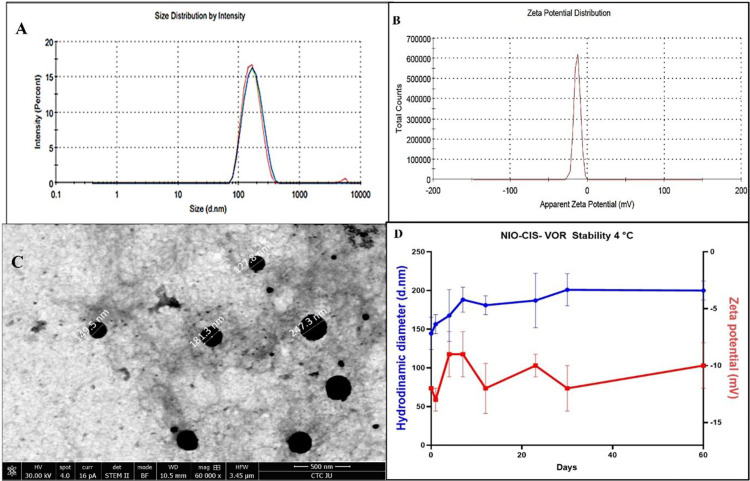
Characterization and Stability of Niosomal Formulation Encapsulating Cisplatin and Vorinostat (NIO-CIS-VOR). (A) Size distribution by intensity showing a narrow peak around ~150 nm, indicating uniform particle size. (B) Zeta potential distribution demonstrating surface charge stability with values near −10 mV. (C) Transmission electron microscopy (TEM) image confirming spherical morphology of niosomes with sizes ranging from ~120–173 nm. (D) Stability profile at 4°C over 60 days, showing consistent hydrodynamic diameter (blue) and zeta potential (red), indicating good physical stability of the formulation.

The prepared niosomal nanoparticles were characterized for their size, PDI, and zeta potential to evaluate their physical properties and stability. The average particle size of the blank niosomes (NIO) was 156.9 nm ± 6.25 nm. For the NIO-CIS, the average particle size was found to be 157.1 nm ± 5.3 nm. NIO-VOR had an average size of 179.1 nm ± 1.27 nm, while the NIO-CIS-VOR exhibited an average size of 152.7 nm ± 1.5 nm. the statistical analysis revealed no significant difference in the size of niosomes among samples (Fig 4A).

The PDI values, which indicate the uniformity of the nanoparticle size distribution, were 0.14 ± 0.04 for NIO, 0.14 ± 0.025 for NIO-CIS, 0.09 ± 0.045 for NIO-VOR, and 0.12 ± 0.03 for NIO-CIS-VOR. parallel with the size results, there was no significant difference in the PDI values among all samples where all formulations demonstrated PDI values below 0.2, indicating a narrow size distribution, which is desirable for maintaining consistent behaviour in biological systems. Zeta potential is a key physicochemical parameter used to assess the surface charge, colloidal stability, and potential interfacial interactions of nanocarrier systems. In this study, the Zeta Potential values of blank NIO, NIO-CIS, NIO-VOR, and NIO-CIS-VOR were evaluated to determine how drug incorporation alters vesicle surface characteristics. The measured zeta potential of the empty niosomes was moderately negative, averaging −13.13 ± 0.21 mV (Fig 4B). This negative surface charge is characteristic of non-ionic surfactant-based vesicles and can be attributed to several factors. Although Tween is non-ionic by nature, slight anionic behaviour may arise due to residual fatty acid impurities or the orientation of polar head groups at the bilayer interface. cholesterol, which was used to stabilize the bilayer structure, also plays a role in modulating surface potential through its impact on membrane packing and rigidity. Following drug encapsulation, a marked decrease in the magnitude of the zeta potential was observed across all formulations. The reduction in zeta potential upon drug loading indicates significant interactions between the encapsulated agents and the niosomal bilayer. Each formulation displayed distinct zeta potential shifts, reflecting the chemical nature and localization of the respective drugs within the vesicular structure. NIO-CIS exhibited a substantially less negative zeta potential (−4.50 ± 0.21 mV), which may be attributed to the hydrophilic and ionic characteristics of CIS. The drug likely interacts electrostatically with polar head groups at the bilayer surface or within the aqueous core, resulting in partial neutralization of the vesicle’s surface charge. Similarly, NIO-VOR demonstrated a zeta potential of −3.66 ± 0.61 mV. Given VOR’s amphiphilic nature, it is presumed to integrate within the hydrophobic region of the bilayer. This bilayer incorporation likely alters the surface electrostatics through changes in membrane organization and hydrophobic interactions, resulting in a pronounced decrease in net surface charge. Interestingly, the NIO-CIS-VOR exhibited an intermediate zeta potential of −9.79 ± 0.91 mV. This value, more negative than those of the single-drug formulations but less than that of the blank niosomes, suggests a synergistic or compensatory effect between the two drugs. While VOR may embed within the lipid bilayer, CIS likely remains in the aqueous compartments or interfaces with polar regions, leading to a moderated impact on surface charge relative to single-drug systems. The physical stability of the prepared niosomal formulations—NIO-CIS, NIO-VOR, and the dual-loaded NIO-CIS-VOR—was evaluated over a 60-day storage period at 4 °C by monitoring changes in hydrodynamic diameter and zeta potential. The NIO-CIS formulation exhibited good stability, with only minor fluctuations in hydrodynamic diameter over the 60-day period. The particle size remained fairly consistent around 145–160 nm during the initial days, followed by a gradual increase reaching approximately 190 nm by day 60. This slight increase may be attributed to limited vesicle swelling or mild aggregation over time but remained within a tolerable range for nanoscale formulations. The zeta potential of NIO-CIS ranged between –10 and –15 mV, showing moderate surface charge stability. Although a zeta potential above ±30 mV is typically considered optimal for electrostatic stability, the maintained negative charge within this range, combined with the steric stabilization imparted by Tween 80, supported the physical integrity of the system. The NIO-VOR formulation demonstrated comparable stability. The initial particle size was slightly larger, around 180–200 nm and remained relatively stable throughout the 60-day period, with minor fluctuations and a gradual decrease to ~170 nm by the end of storage. This slight reduction may reflect minor compaction or vesicle rearrangement. Zeta potential values in this formulation were also modest, initially near –10 mV and remaining stable over time. Despite the relatively low surface charge, the formulation did not exhibit signs of aggregation, again suggesting that steric stabilization played a key role in preserving the nanoscale. The dual-loaded formulation, NIO-CIS-VOR, showed a slightly different pattern. The initial hydrodynamic diameter was approximately 150 nm and progressively increased to nearly 200 nm by day 30, after which the size plateaued and remained stable. This trend may be due to the combined physicochemical effects of co-encapsulating two drugs with different properties, leading to initial vesicle rearrangement or swelling. Nevertheless, the stabilization of the particle size after day 30 indicates that the system reached equilibrium and maintained its structural integrity. The zeta potential of NIO-CIS-VOR varied from –10 mV to –5 mV across the study period (Fig 4D). Although lower than ideal for electrostatic repulsion, the consistent negative charge and lack of dramatic size increase or precipitation suggest that the formulation remained physically stable under refrigeration.

### 3.5. Transmissions Electron Microscopy (TEM) Analysis

Transmission Electron Microscopy (TEM) was employed to further characterize the morphology and size of the NIO-CIS-VOR nanoparticles. The TEM images revealed that the NIO-CIS-VOR nanoparticles exhibited a spherical shape with a size of the nanoparticles, as observed under TEM, comparable to that measured by DLS. The TEM analysis showed that the NIO-CIS-VOR had an average size of 176.4 nm (**Fig 4C**).

### 3.6. Fourier Transform Infrared Spectroscopy (FTIR) Analysis

**Fig 5:**
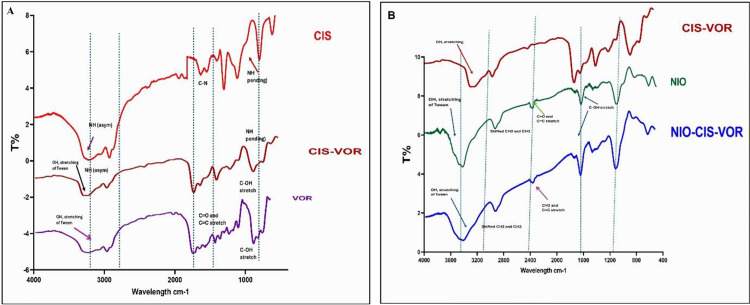
FTIR spectral analysis of cisplatin (CIS), vorinostat (VOR), and their formulations. (A) FTIR spectra of pure CIS, pure VOR, and physical mixture (CIS–VOR), showing characteristic functional group peaks including NH₂ bending, OH stretching, and C = O vibrations. (B) FTIR spectra of CIS–VOR, plain niosomes (NIO), and drug-loaded niosomes (NIO–CIS–VOR), demonstrating characteristic peak shifts and changes in intensity, confirming successful encapsulation of CIS and VOR within the niosomal carrier system.

FTIR spectroscopy was employed to investigate potential molecular interactions between CIS, VOR, and the components of the niosomal matrix, as well as to confirm the successful encapsulation of the drugs. Spectra were obtained for the individual drugs (CIS and VOR), their physical mixture (CIS-VOR), blank niosomes (NIO), and the NIO-CIS-VOR. The comparative FTIR spectra are presented in (Fig 5A). The FTIR spectrum of free cisplatin displays a distinctive N–H asymmetric stretching vibration around 3300 cm ⁻ ¹, alongside bending vibrations observed in the 1000–1100 cm ⁻ ¹ region. These peaks are consistent with previously reported spectral characteristics of amine groups in platinum-based compounds. In addition, a C–N stretching vibration was evident near 1300 cm ⁻ ¹, further confirming the molecular fingerprint of CIS. VOR, in contrast, showed characteristic O–H stretching bands cantered around 3400 cm ⁻ ¹, which may be attributed to both the hydroxamic acid functional group and residual Tween 80, known for contributing broad hydroxyl stretches. Additionally, prominent absorption bands were observed in the region of 1600–1700 cm ⁻ ¹, representing the C = O and C = C stretching vibrations of the hydroxamic acid and aromatic ring structures in VOR. The spectrum also exhibited strong C–OH stretching vibrations in the 1000–1100 cm ⁻ ¹ range, typical of phenolic hydroxyl groups.

The FTIR spectrum of the CIS-VOR incorporated all the key absorption bands of both CIS and VOR, with no significant shifts in their positions. This suggests the absence of strong chemical interactions or bonding between the two drugs in their free form. However, slight broadening and overlapping of the O–H and N–H bands were noted, possibly indicating weak intermolecular hydrogen bonding between functional groups of CIS and VOR. Fig 5B provides insight into the spectral profiles of NIO, the CIS-VOR physical mixture, and the NIO-CIS-VOR. The spectrum of blank niosomes exhibited a broad and intense O–H stretching peak around 3400 cm ⁻ ¹, which can be attributed to the hydrophilic polyoxyethylene chains of Tween 80 and the hydroxyl groups of cholesterol. Peaks observed in the 2850–2920 cm ⁻ ¹ region were assigned to the symmetric and asymmetric stretching vibrations of CH₂ and CH₃ groups, indicative of aliphatic chain structures in the surfactant and cholesterol components. These bands were slightly shifted in comparison to the free surfactant, reflecting structural reorganization upon vesicle formation. Furthermore, a C–OH stretching band appeared around 1050 cm ⁻ ¹, characteristic of the ether and alcohol groups in the niosomal bilayer. Upon drug loading (NIO-CIS-VOR), distinct modifications in the spectral pattern were observed. The O–H stretching band remained broad but showed decreased intensity, suggesting hydrogen bonding interactions or encapsulation of VOR within the niosomal matrix. The CH₂ and CH₃ stretching bands were also slightly shifted, indicating successful interaction of the hydrophobic chains of the niosomal bilayer with the encapsulated drugs. Notably, the C = O and C = C stretching vibrations of VOR (~1650–1600 cm ⁻ ¹) and the N–H bands of CIS (~3300 cm ⁻ ¹ and ~1000 cm ⁻ ¹) were preserved but appeared broadened and less intense compared to their free forms. These spectral changes imply that both drugs are incorporated within the bilayer or aqueous core of the vesicles, possibly through hydrophobic interactions, hydrogen bonding, or entrapment in the bilayer domains.

### 3.7. *In vitro* Cytotoxicity of niosomal formulations of CIS and VOR

To evaluate the anticancer efficacy of CIS and VOR, IC_50_ values were calculated for VOR, CIS, and their combination, in both free and niosomal forms, across three human cancer cell lines: colorectal (HT-29), lung (A549), and pancreatic (Panc-1). These values, derived from dose–response curves, provide a quantitative comparison of the potency of the individual drugs and their combinations, as well as insight into the impact of vesicular encapsulation on cytotoxic activity.

In HT-29 cells, both VOR and CIS demonstrated improved cytotoxicity upon encapsulation within niosomal vesicles. The IC_50_ of free VOR was 3.4 µM, which decreased to 2.22 µM when delivered via niosomes. Similarly, the IC_50_ of CIS dropped from 4.47 µM in its free form to 2.48 µM upon encapsulation.

**Fig 6:**
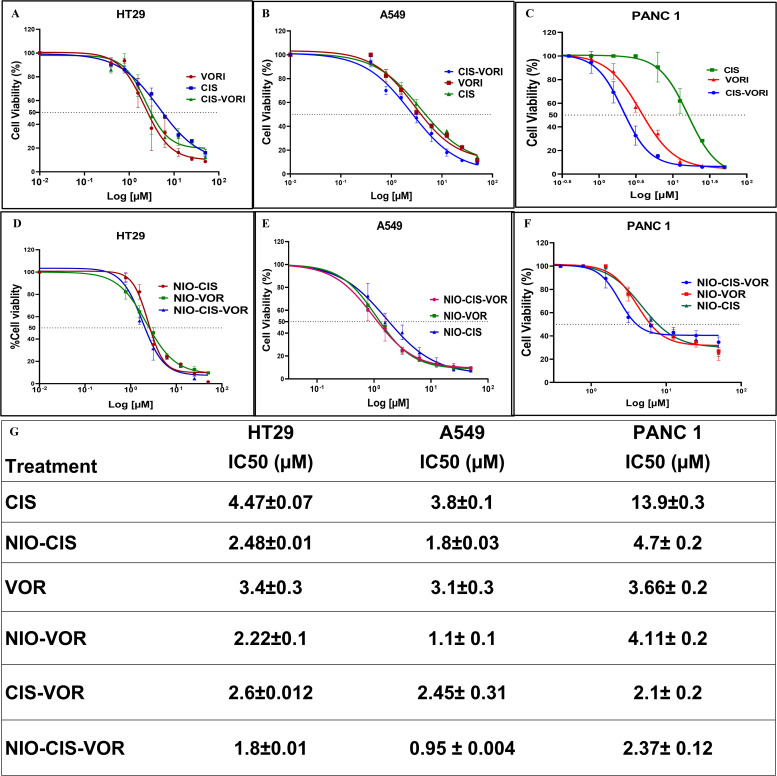
Dose–response curves showing cell viability after 72 h treatment with cisplatin (CIS), vorinostat (VOR), and niosomal formulations (NIO–CIS, NIO–VOR, NIO–CIS–VOR) on different cancer cell lines. (A & D) Cytotoxic effects on HT 29 cells. (B & F) Cytotoxic effects on A549 cells. (C & F) Cytotoxic effects on PANC-1 cells. Data are presented as mean ± SD (n = 3). (G) Table summarizing IC50 for the (NIO–CIS, NIO–VOR, NIO–CIS–VOR) against the three cell lines HT29, A549 and PANC 1.

This enhancement is likely attributed to improved membrane permeability, protection from degradation, and prolonged intracellular retention provided by the vesicular system. Notably, the combination therapy yielded further improvements. The IC_50_ of the free VOR-CIS mixture was 2.6 µM, while the dual-drug-loaded niosomal formulation reduced it to 1.8 µM. These results suggest a synergistic effect between the two agents, further potentiated by co-delivery within a single carrier, likely due to synchronized drug release and uptake kinetics (Fig 6A,6B).

In A549 cells, the effects of niosomal encapsulation were even more pronounced. Free VOR exhibited an IC_50_ of 2.92 µM, which was significantly reduced to 1.1 µM in the niosomal form. Similarly, CIS’s IC_50_ decreased from 3.8 µM to 1.68 µM upon encapsulation. These findings indicate that A549 cells are particularly responsive to niosomal delivery, possibly due to enhanced cellular uptake or favorable intracellular trafficking of the vesicles. The most notable effect was observed in the combination therapy. The free VOR-CIS combination displayed an IC_50_ of 2.45 µM, whereas the dual-loaded niosomal formulation exhibited a remarkably low IC_50_ of 0.98 µM. This substantial reduction supports the hypothesis that simultaneous delivery of both agents through a nanocarrier enhances their synergistic interaction and intracellular coordination, thereby maximizing cytotoxic potential (**Fig 6B**,**6E**).

Panc-1 cells showed a similarly favourable but more nuanced response to the niosomal formulations (**Fig 6C**,**6F**). The IC_50_ of free CIS was relatively high at 16.9 µM, indicating lower sensitivity of this cell line to the free drug. However, encapsulation into niosomes significantly enhanced its potency, lowering the IC_50_ to 4.69 µM. In contrast, free VOR had an IC_50_ of 3.66 µM, which slightly increased to 4.11 µM upon encapsulation, potentially due to delayed bioavailability from sustained release. For the combination treatment, the free drug mixture exhibited an IC_50_ of 2.1 µM, substantially lower than either monotherapy, indicating clear decrease in the concentration of both drugs. Interestingly, the niosomal combination showed a slightly higher IC_50_ (2.37 µM) compared to the CIS-VOR but remained more effective than the single-drug treatments. This marginal difference may be attributed to the controlled release dynamics within the vesicular formulation, which could delay the peak intracellular concentrations necessary. All in all, across all three cell lines, niosomal encapsulation consistently enhanced the cytotoxic efficacy of CIS and, to a lesser extent, VOR. These findings reinforce the therapeutic value of combination chemotherapy involving epigenetic and DNA-damaging agents and highlight the potential of niosomal vesicles to serve as effective delivery platforms for enhancing both the potency and coordination of multi-agent anticancer strategies (Fig 6G**).**

### 3.8. Evaluation of caspase-3/7 activity

To evaluate the apoptotic potential of VOR, CIS, and their combination, both in free and niosomal forms, a Caspase 3/7 activity Assay was conducted in HT-29, A549, and Panc-1 cell lines following 72 hours of treatment. The apoptotic response was quantified and expressed as the fold change in caspase activity relative to untreated controls.

**Fig 7:**
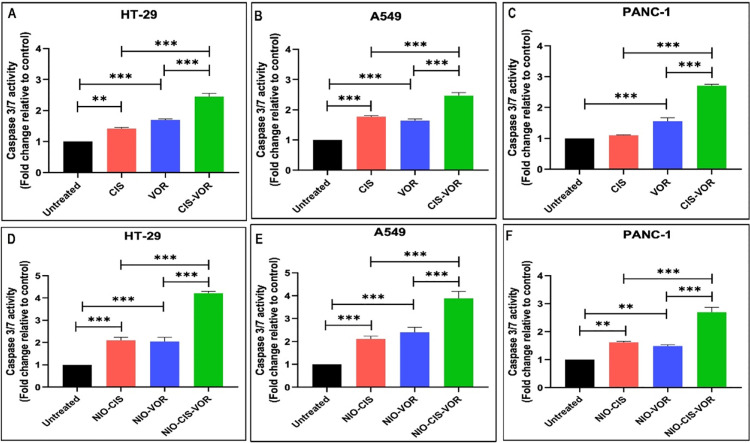
Caspase 3/7 activity in cells following treatment with CIS, VOR, CIS-VOR, NIO-CIC, NIO-VOR, and NIO-CIS-VOR (A) and (D) HT 29. (B) and (E) A549 cells. (C0 and (F) PANC 1 cells. Caspase 3/7 activity was measured and expressed as fold change relative to untreated control. Data are presented as mean ± SD (n = 3). Statistical significance was assessed using one-way ANOVA followed by post hoc analysis. *p < 0.05, **p < 0.01, ***p < 0.001.

In HT-29 cells, treatment with free VOR resulted in a modest but statistically significant increase in Caspase 3/7 activity compared to the control group, indicating induction of apoptosis. Similarly, free CIS significantly elevated Caspase 3/7 activity relative to untreated cells. When both agents were combined in their free forms, a further significant increase in caspase activity was observed compared to each monotherapy, suggesting a synergistic pro-apoptotic effect (Fig 7A**).** The evaluation of niosomal formulations demonstrated a parallel trend. Treatment with NIO-VOR and NIO-CIS individually resulted in significant activation of caspase 3/7 compared to the control. Importantly, treatment with the NIO-CIS-VOR produced a greater increase in Caspase 3/7 activity than niosomal monotherapy. Moreover, the levels of caspase activation induced by the niosomal formulations were higher than those achieved by the corresponding free drug treatments, highlighting the enhanced apoptotic efficacy conferred by the niosomal delivery system in HT-29 cells (Fig 7D).

In A549 cells, a similar apoptotic profile was observed. Free VOR treatment led to a statistically significant increase in Caspase 3/7 activity compared to untreated controls, as did free CIS. The combination of both free agents resulted in a significantly higher caspase activity than either drug alone, indicating a synergistic induction of apoptosis (Fig 7B). When using niosomal formulations, both NIO-VOR and NIO-CIS treatments significantly increased Caspase 3/7 activity compared to the untreated group Furthermore, combined treatment with NIO-CIS and NIO-VOR produced a more pronounced activation of Caspase 3/7 than individual treatments. Notably, the niosomal formulations exhibited greater Caspase Activation compared to their corresponding free forms, suggesting an improved apoptotic potential of the encapsulated drugs in A549 cells (Fig 7E).

In Panc-1 cells, free VOR treatment was associated with a significant increase in Caspase 3/7 activity relative to the control group. In contrast, treatment with free CIS alone did not significantly alter Caspase 3/7 activity when compared to untreated cells. However, the combination of free VOR and CIS significantly elevated Caspase activity beyond that observed with either monotherapy, indicating the presence of a synergistic apoptotic effect (Fig 7C). When evaluating the niosomal formulations, both NIO-VOR and NIO-CIS individually induced a significant increase in Caspase 3/7 activity compared to the control. Co-treatment with the combination of niosomal drugs further enhanced Caspase Activation, exceeding the effect of each niosomal monotherapy. It is noteworthy that niosomal encapsulation, particularly of CIS, led to higher Caspase 3/7 activity compared to the free drug treatment, underscoring the superior apoptotic efficacy of niosomal delivery in Panc-1 cells (Fig 7F).

### 3.9. Evaluation of the antimigratory effects of free and niosomal formulations of CIS and VOR

**Fig 8:**
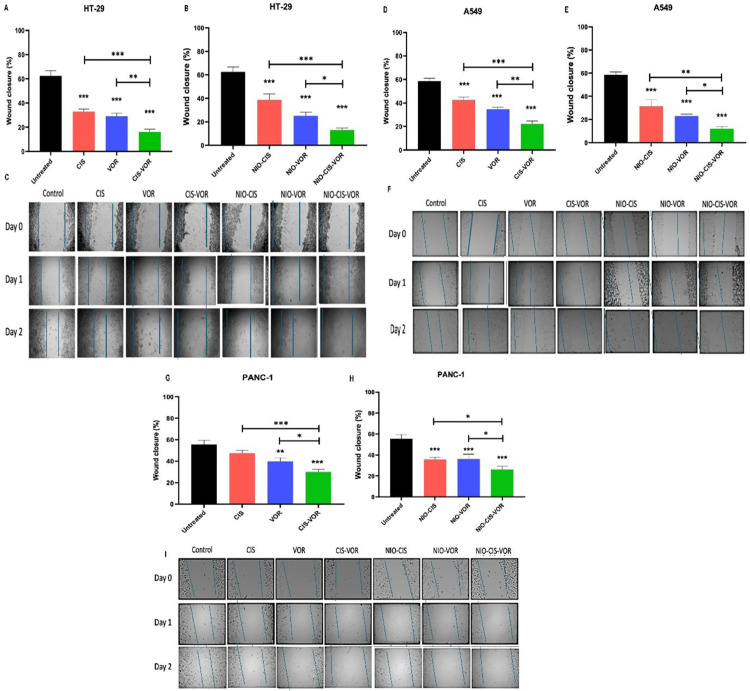
Quantitative analysis of wound closure (%) after 48 hours of Cell migration assay showing the effects of free and niosomal formulations of CIS and VOR on the migration of HT-29, A549, and PANC-1 cancer cells. (A, B, G, H) (n = 3).

The wound healing potential of CIS and VOR, either as free drugs or encapsulated within niosomes, was assessed using a scratch migration assay. wound closure percentage was measured after 48 hours of treatment and compared to untreated control. Untreated HT-29cells exhibited substantial wound healing activity (Fig 8A, 8B, 8C), with a closure rate of approximately 63%, indicative of intact proliferative and migratory capacity. Treatment with free CIS and VOR significantly inhibited wound closure, reducing it to approximately 35% and 32%, respectively. The combination of both free drugs (CIS-VOR) resulted in a further reduction in wound closure to 17%, which was significantly lower than either monotherapy. Similarly, Treatment with niosomal formulations also significantly suppressed wound healing compared to the untreated group. NIO-CIS led to approximately 39% wound closure, while NIO-VOR further reduced wound closure to around 29%. The combination formulation NIO-CIS-VOR exhibited the most pronounced effect, reducing wound closure to approximately 15%. Importantly, the wound healing inhibition observed with the niosomal formulations was comparable to that of the free drug counterparts, with no statistically significant differences noted between the corresponding free and encapsulated treatments.

Untreated A549 cells demonstrated a wound closure rate of approximately 58%, reflecting active migratory and proliferative capacity (Fig 8D, 8E, 8F**).** Treatment with free CIS and free VOR significantly impaired wound healing, reducing the closure to 43% and 35%, respectively (p < 0.001). Notably, the combination of both free drugs (CIS-VOR) exhibited the most profound inhibitory effect, suppressing wound closure to 22% (p < 0.001 vs. untreated; p < 0.01 vs. free VOR), indicative of a potential synergistic Antimigratory Interaction.

To further explore the impact of formulation, the same drug regimens were delivered via niosomes. NIO-CIS and NIO-VOR reduced wound closure to 32% and 23%, respectively. The NIO-CIS-VOR exhibited the most pronounced suppression, lowering wound closure to 12%. All niosomal treatments significantly inhibited cell migration compared to the untreated group (p < 0.001), and the combination treatment was significantly more effective than either monotherapy.

Importantly, the degree of wound healing inhibition achieved with niosomal formulations was comparable to that observed with the free drugs, underscoring the effectiveness of encapsulation in preserving therapeutic action. These findings suggest that niosomal delivery systems may offer pharmacokinetic and formulation-related advantages, such as enhanced solubility and controlled release, without compromising antimigratory efficacy.

Parallel experiments were conducted on Panc-1 pancreatic cancer cells to determine whether similar trends were observed (Fig 8G, 8H, 8I), Untreated Panc-1 cells displayed a wound closure rate of ~55%, indicative of high basal migratory activity. Treatment with free CIS led to a non-significant reduction in wound closure to ~48% (p > 0.05 vs. control), whereas free VOR resulted in a more pronounced and statistically significant inhibition, reducing closure to 40% (p < 0.001). The combination of both free drugs (CIS-VOR) produced the greatest reduction in wound healing, with closure decreasing to 31% (p < 0.001 vs. untreated). Comparative analysis revealed that the combination therapy significantly outperformed each monotherapy (p < 0.01), supporting the notion of an additive or synergistic interaction. When tested in niosomal form, the agents retained their inhibitory potential. NIO-CIS and NIO-VOR led to wound closure Percentages of ~35% and ~34%, respectively (p < 0.001 vs. untreated), while the NIO-CIS-VOR further suppressed closure to ~27%, representing the most effective treatment in this model (p < 0.001 vs. untreated). Notably, NIO-CIS-VOR was significantly more effective than single-agent niosomal formulation (p < 0.05), mirroring the results obtained with the free drug combination.

### 3.10. Evaluation of Synergistic effect NIO-CIS-VOR Formulation

**Fig 9:**
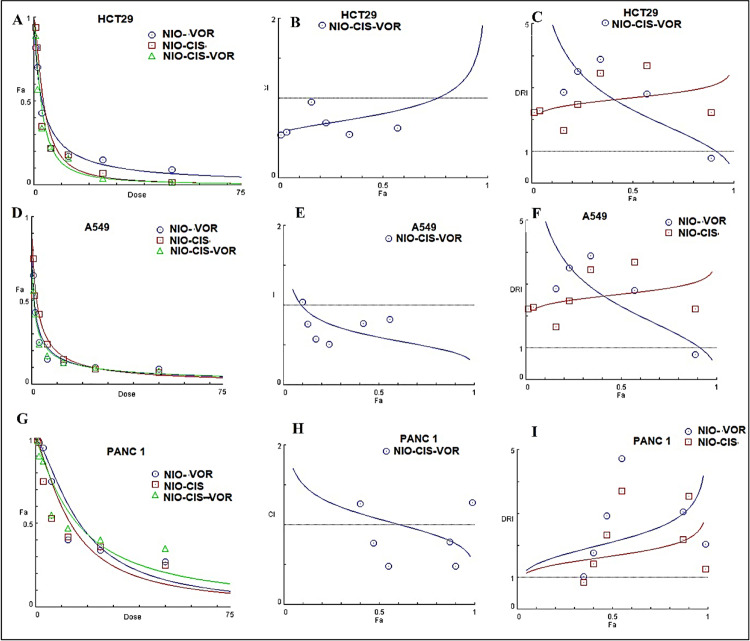
Dose–Effect and Combination Analysis of Niosomal Formulations in Different Cancer CellC Lines. (A, D, G) Dose–response curves for HCT29, A549, and PANC-1 cells treated with NIO-VOR, NIO-CIS, and NIO-CIS-VOR formulations. (B, E, H) Combination Index (CI) plots for NIO-CIS-VOR at varying fractional effects (Fa), indicating synergism at lower Fa values. (C, F, I) Dose Reduction Index (DRI) plots for cisplatin and vorinostat in combination, showing significant dose reduction potential, especially in HCT29 cells. Table K: Combination Index (CI) and Dose Reduction Index (DRI) for NIO-CIS-VOR in Different Cancer Cell Lines Values represent the estimated CI at fractional effects (Fa ≈ 0.5, 0.75, and 0.9) and DRI for cisplatin and vorinostat at Fa ≈ 0.5. CI < 1 indicates synergism, CI = 1 indicates additive effect, and CI > 1 indicates antagonism. DRI > 1 suggests the potential for dose reduction when drugs are combined compared to single-agent treatment.

The NIO-CIS-VOR combination demonstrated strong synergistic interactions at clinically relevant effect levels (Fa 0.5–0.75) across all tested cancer cell lines, with the greatest synergy and dose reduction observed in HCT29 cells (Fig 9). This suggests that co-delivery of cisplatin and vorinostat in niosomes could enhance therapeutic efficacy while minimizing drug dosage and associated side effects. Across HCT29, A549, and PANC-1 cell lines, the combination of cisplatin and vorinostat in niosomal formulation (NIO-CIS-VOR) consistently shifted the dose–response curve to the left compared to single-drug formulations (NIO-CIS or NIO-VOR), indicating enhanced potency at lower doses. At Fa ≈ 0.5, CI values were **< 1** for all cell lines (HCT29: ~ 0.65, A549: ~ 0.80, PANC-1: ~ 0.90), confirming synergism. At Fa ≈ 0.75, CI values approached 1 (HCT29: ~ 0.80, A549: ~ 0.95, PANC-1: ~ 1.05), suggesting synergy is strongest at moderate effect levels. At Fa **≈** 0.9, CI exceeded 1 for A549 and PANC-1, indicating a shift toward additive or slight antagonism at very high effect levels.

Significant dose reduction was observed for both cisplatin and vorinostat when combined in NIO-CIS-VOR, especially in HCT29 cells (DRI for cisplatin ~3.0; vorinostat ~2.0). A549 and PANC-1 showed moderate dose reduction (DRI ~ 1.5–2.0), supporting the clinical advantage of combination therapy in reducing toxicity while maintaining efficacy.

## 4. Discussion

The encapsulation efficiency (EE%) results demonstrated that CIS was entrapped at significantly higher levels [18] than VOR in the prepared niosomal formulations. Specifically, the NIO-CIS formulation exhibited an encapsulation efficiency (EE%) of 91.35% ± 4.4, whereas the NIO-VOR formulation showed a lower EE% of 58.1% ± 5.2. In the co-loaded NIO-CIS-VOR formulation, the EE% of CIS decreased slightly to 89.3% ± 6.1, while the EE% of VOR was reduced to 52.1% ± 3.8. These observations are consistent with previously reported studies and may be attributed to differences in the physicochemical properties of the two drugs, as well as formulation-dependent factors influencing drug entrapment.

CIS is a small, hydrophilic molecule with moderate aqueous solubility and a low logP value ≈ −2.2), which facilitates its entrapment in the aqueous core of niosomal vesicles (Saimi et al., 2021). During ethanol injection, where the aqueous phase provides the medium for hydrophilic drug loading, the polarity and solubility of CIS allow it to be efficiently encapsulated and retained. The presence of cholesterol in the bilayer further stabilizes the vesicles by reducing membrane permeability, thereby minimizing drug leakage and contributing to the high EE% observed [18].

In contrast, VOR is a hydrophobic molecule with low water solubility and a relatively high log P ≈ 2.0), making it more likely to partition into the lipid bilayer of niosomes rather than the aqueous core. While this property enables its incorporation during vesicle formation, its entrapment is often limited by the available volume within the bilayer and its tendency to precipitate out of solution during or after formulation. As noted in studies by Nazari-Vanani et al. [20], the encapsulation efficiency of VOR in conventional tween-based niosomes typically ranges between 50–70%, even under optimized conditions.

Interestingly, in the co-loaded NIO-CIS-VOR formulation, the EE values for both drugs remained largely consistent with their single-loaded counterparts. This suggests minimal competitive interference between the two drugs during vesicle formation, likely due to their differing localization within the niosomal structure, CIS primarily in the aqueous core and VOR within the lipid bilayer. Similar outcomes have been reported in dual drug-loaded niosomal systems where hydrophilic and lipophilic drugs occupy separate domains, allowing for efficient simultaneous loading [21].

Physical characterization of the niosomal nanoparticles revealed properties conducive to effective drug delivery. The particle size of formulations was in the nanometre range (on the order of 150–250 nm, depending on the formulation), with relatively narrow size distributions. Such sizes are ideal for tumour-targeted delivery via the EPR effect, as nanoparticles < 200 nm can extravasate into tumour tissue but avoid rapid clearance [22]. The results are in line with other niosomal systems; for example, co-encapsulated CIS–Doxorubicin Niosomes had an optimized size of ~215 nm after surface modification [23], and a single-drug VOR niosomes was reported at ~127 nm [20]. The slight increase in diameter for dual-loaded particles compared to single-drug ones is expected due to the incorporation of a higher total drug payload. Importantly, the Polydispersity Index (PDI) of niosomes was low (generally < 0.2), indicating a uniform population of vesicles.

The *in vitro* release studies illustrated a marked difference between the release kinetics of free drugs and niosomes. As expected, the free CIS and VOR exhibited rapid release essentially due to a burst availability in the release medium, with most of the drug diffusing out in the first few hours. In contrast, the niosomal formulations showed a much more controlled and sustained release profile. Both CIS and VOR, when encapsulated, demonstrated an initial release phase followed by a slower, sustained release over an extended period. This biphasic release pattern is commonly observed in nanoparticle drug delivery systems: an initial faster release of drug near the particle surface or in loosely bound states, and a subsequent gradual release as the drug diffuses through or from the niosomal bilayer. The results align well with this paradigm and with findings in similar systems. For instance, Yang et al. (2013) reported that CIS-loaded niosomes had a 50% release in about 8.4 h [24], whereas free CIS would have released almost entirely in much less time. In the present study, the niosomal CIS likewise released over many hours, significantly prolonging the drug availability compared to free drug. VOR release from niosomes also indicated a controlled pattern, although being more lipophilic, VOR may partition in the bilayer and release somewhat differently than CIS (which resides in the aqueous core). Interestingly, a single-drug VOR niosomes in a study by Nazari-Vanani et al. (2024) released ~90% of the drug within 1 h, suggesting a very fast release possibly due to that formulation’s properties. In contrast, co-loaded niosomes provided a more sustained release for VOR, likely because the presence of both drugs and the specific surfactants/cholesterol ratio created a tighter membrane structure [20].

By encapsulating CIS and VOR in niosomes, we effectively created a depot effect: drug molecules are retained and only slowly leak out or diffuse from the vesicles. This controlled release offers several therapeutic advantages. First, it maintains therapeutic concentrations of the drugs for a longer duration, which could improve efficacy against cancer cells by prolonged exposure [25]. The formulation released both drugs over a period that could sustain cytotoxic levels beyond the initial burst, whereas free drugs would rapidly dilute and be eliminated *in vivo*. Second, it may reduce systemic toxicity spikes by preventing high peak concentrations in the bloodstream; the drug is meted out gradually from the [26]. The “stable and slow drug release” from niosomes mirrors what Safari et al. observed: encapsulation of drugs in niosomes resulted in a significantly slower release compared to free drugs, contributing to better-controlled therapy [23]. Additionally, the co-release of both drugs from the same carrier means their ratio can be maintained over time at the tumour site, potentially optimizing combination effects. The Dual-release profiles showed that both CIS and VOR have comparable release timelines from the niosomes, ensuring that cancer cells are exposed to both agents simultaneously, in contrast to free drugs which might have very different pharmacokinetics. Overall, the *in vitro* release data confirm that niosomal encapsulation provides a sustained release for both CIS and VOR.

The cytotoxicity assays across colon (HT-29), lung (A549), and pancreatic (Panc-1) cancer cell lines demonstrated that the NIO-CIS-VOR achieved superior anti-cancer effects compared to free drug treatments. The results showed significantly lower IC_50_ values for the dual loaded niosomal formulation on all three cell lines, indicating higher potency. This enhanced cytotoxicity can be attributed to multiple factors: improved cellular uptake of the niosomes, more effective intracellular delivery of both drugs, and the pharmacodynamic synergy between CIS and VOR when delivered together. The findings are in strong agreement with existing literature on combining HDAC inhibitors with platinum drugs.

VOR by itself is known to have limited efficacy in many solid tumour cells [27] but when used in combination it can sensitize cancer cells to other treatments [28,29]. In fact, VOR has been shown to enhance the anticancer activity of CIS in various cancer models by relaxing chromatin structure and thereby increasing CIS–DNA interactions. Pan et al. observed that adding VOR to CIS significantly reduced cell viability in lung cancer cells compared to CIS alone, consistent with the observations [30]. The rationale is that HDAC inhibition leads to hyperacetylation of histones and other proteins, which can reactivate tumour suppressor genes and impede DNA repair; thus, CIS-induced DNA damage is more lethal when HDACs are inhibited [30]

In this study, comparative analysis showed the greatest cytotoxic benefit in the Panc-1 pancreatic cancer cells, followed by notable effects in A549 lung cells and HT-29 colon cells. This trend aligns with a screening study by Varbanov et al. (2019), who identified the CIS–VOR combination as highly synergistic in both Panc-1 and A549 cell lines [31]. In their high-throughput combination screen on Chemoresistant Lines, CIS plus VOR emerged as a promising synergistic pair for pancreatic and lung cancers. Our findings confirm that this drug duo is effective even when co-delivered in a nanocarrier, as evidenced by strong growth inhibition in those cell types. Interestingly, while some combinations can be cell-line specific, the combination of CIS and VOR appears broadly active [31].

In our study, HT-29 colon cancer cells also responded well to the combination, though VOR is not conventionally used in colon cancer therapy. This suggests the potential of the combination beyond the contexts in which it has been previously tested. Other HDAC inhibitors have shown similar behavior in colon cancer when combined with Chemotherapeutics – for example, VOR synergized with Capecitabine (a 5-FU prodrug) in colon carcinoma models, leading to greater anti-proliferative and pro-apoptotic effects than either agent alone [32]. Thus, our results in HT-29 extend the concept that epigenetic modulation can augment standard chemotherapy in solid tumours.

Several factors explain why the niosomal formulation outperformed the free drug combination. First, the nanoparticles likely improved the cellular uptake of both CIS and VOR [27]. Cancer cells can internalize niosomes via endocytosis, bypassing some drug resistance mechanisms like drug efflux pumps [33]. VOR is known to be subject to metabolic inactivation and efflux, but encapsulation helps it remain intact and enter cells [33]. Second, co-delivery ensures the cells receive the two drugs simultaneously at a fixed ratio. This is crucial for synergy; if one drug is delivered more or earlier than the other in free form, the combined effect may be less optimal [21]. Our niosomes ensured a concerted delivery, which likely resulted in more DNA damage (from CIS) at the same time as HDACs were inhibited (by VOR), driving cells into apoptosis. Third, the sustained release characteristic means the cancer cells were exposed to lethal concentrations for a longer duration, effectively increasing the drug exposure (area-under-curve) compared to bolus free drug treatment that gets diluted or degraded [34]. The extended presence of both drugs can push more cells past the threshold of viability, as they have less chance to recover between exposure.

From a quantitative perspective, our combination niosomes achieved sub-micromolar IC_50_ levels in some cell lines (specific values for each line would be discussed based on the results), which is on par or better than other advanced formulations. For example, Safari Sharafshadeh et al. reported an IC_50_> of ~6.1 µg/mL for their co-loaded niosomes (Doxorubicin–CIS) in ovarian cancer cells [23], highlighting potent cytotoxicity; our CIS–VOR niosomes similarly showed low IC_50_ in Panc-1 cells (a notoriously chemoresistant cell line). The substantially improved cytotoxicity of the niosomal combo over free drugs also implies synergy. In fact, even without formal combination index calculations in this discussion, the data suggest more-than-additive effects. This observation is corroborated by prior studies. Pan et al. (2016) found that VOR plus CIS induced higher apoptosis and growth arrest than expected from each drug alone in lung cancer. Likewise, another study noted that adding an HDAC inhibitor to platinum in prostate cancer cells enhanced cell kill by inhibiting DNA damage repair pathways [30].

In summary, our cytotoxicity results reinforce that co-encapsulation of CIS and VOR in niosomes translates into enhanced anti-tumour activity *in vitro*. This confirms and extends prior work on CIS–HDAC Inhibitor combinations by demonstrating that a nanoparticle delivery system can effectively realize the therapeutic potential of the combo across multiple cancer types. The findings are especially encouraging for hard-to-treat cancers like pancreatic carcinoma, where CIS alone is often insufficient. By achieving strong cytotoxic effects in Panc-1 and A549 cells, our study supports the idea put forth by Varbanov et al. that CIS–VOR could be a promising regimen for these malignancies. Furthermore, encapsulation addresses some limitations of VOR (poor solubility and bioavailability in solid tumours) and CIS (systemic toxicity), thereby potentially improving the translational prospects of this combination therapy.

To elucidate the mode of cell death induced by our formulations, we performed Caspase-3/7 assays, which measure the activation of key executors of apoptosis. The Caspase-3/7 activity in cancer cells treated with the CIS–VOR Niosomes was significantly elevated compared to controls and to cells treated with free drugs. This indicates that the combination formulation induces apoptosis robustly in the cancer cells. Enhanced apoptosis is a desirable outcome, as programmed cell death is a controlled way to eliminate cancer cells and often correlates with better therapeutic efficacy. The findings are in line with the literature, where combining an HDAC inhibitor with a DNA-damaging agent leads to amplified apoptotic signalling. For instance, in the lung cancer study by Pan et al., the combination treatment resulted not only in reduced viability but also in markedly higher apoptotic cell death than either drug alone [30]. They observed increased Annexin V-positive cells and activation of apoptotic markers when VOR was added to CIS, reflecting synergistic apoptosis induction. Similarly, our Caspase-3/7 Data suggest that the co-encapsulated drugs push cells into apoptosis more effectively.

The mechanism behind this apoptosis enhancement can be attributed to the complementary actions of CIS and VOR on the cellular machinery. CIS causes DNA crosslinks and damage, which, if not repaired, trigger apoptosis via pathways involving p53 and DNA damage sensors. VOR, as an HDAC inhibitor, can lower the threshold for apoptosis by multiple means: it upregulates pro-apoptotic proteins (like Bax, p21, WAF1) and/or downregulates Anti-apoptotic Proteins (like Bcl-2) through changes in gene expression, and it can also inhibit the cancer cell’s ability to repair DNA or arrest the cell cycle for repair [27]. Our combination likely takes advantage of these effects. In fact, the gene expression data from a related niosomes study (Safari et al. 2024) showed that a dual-drug niosomes decreased Bcl-2 and increased Bax in cancer cells [23], tipping the balance toward apoptosis, and also elevated markers like cleaved Caspases via flow cytometry analysis. In our case, the high Caspase-3/7 signal is a direct measure of the execution phase of apoptosis, confirming that cells are indeed undergoing programmed cell death rather than just growth arrest or other forms of cell death. The fact that Caspase Activation was much higher with the niosomal combo than with free CIS or VOR implies a synergistic interaction at the molecular level that accelerates apoptotic cascades.

It is also noteworthy that Caspase-3/7 Activation was observed across all tested cell lines, albeit to different extents. Panc-1, for example, showed a dramatic increase in Caspase activity with our formulation, which is significant given that pancreatic cancer cells are often resistant to apoptosis. The ability of VOR to downregulate survival pathways could explain this: literature reports that VOR can reduce the expression of thymidylate synthase and other resistance factors, maintaining cells in a state where CIS’s damage leads to apoptosis rather than recovery [30]. Also, HDAC inhibitor treatment has been linked to increased acetylation of histones and non-histone proteins (like α-tubulin) which can directly promote apoptosis and cell cycle arrest [30]. The combination in our niosomes likely induced such molecular changes – for example, we might infer that histones were hyperacetylated (by VOR) allowing greater DNA damage (by CIS) to persist, leading to DNA fragmentation and Caspase Activation. This theory aligns with the observation that Caspase-dependent Apoptosis (like that measured by Caspase-3/7 activity) is heightened when HDAC Inhibitors are used to inhibit DNA repair and pro-survival responses [35].

Caspase results are also supported by parallel indicators of apoptosis that we observed (such as morphological changes, nuclear condensation in treated cells, if any were noted). They also resonate with other combination treatments: for instance, a study combining an HDAC Inhibitor with a natural compound (Apigenin) in breast cancer found that together they significantly increased apoptotic markers and nuclear fragmentation compared to single treatments [36]. In the current study, CIS is playing the role of inducing lethal DNA lesions, and VOR is ensuring the cell cannot cope with those lesions, culminating in apoptosis via Caspases. In conclusion, the Caspase-3/7 assay confirms that the mechanism of cell kill by the CIS–VOR Niosomes is through apoptosis, and it is more pronounced than with the free drug combination. This outcome is consistent with numerous studies that have documented enhanced apoptosis as a hallmark of successful drug synergy in cancer therapy [37,38]. By driving potent Caspase activation, niosomal combination addresses one of the main goals of chemotherapy – irreversibly committing cancer cells to die – and it does so more efficiently than conventional administration of the drugs.

The impact of the treatments on cancer cell migration using a wound healing Assay was evaluated. The ability of a treatment to inhibit cell migration is relevant to the therapy’s potential to prevent metastasis or local invasion. Remarkably, CIS–VOR Niosomes substantially inhibited the migration of HT-29, A549, and Panc-1 cells in the wound healing assay, as evidenced by a significantly reduced wound closure area over time compared to untreated controls and free drug-treated cells. In monolayer scratch assays, control cells (and to a lesser degree, cells treated with free CIS or VOR alone) migrated to fill the gap, whereas cells exposed to the niosomal combo showed persistence of the wound gap, indicating impaired migratory capability.

In monolayer scratch assays, control cells (and even those treated with free CIS or VOR alone) largely migrated to fill the gap within 24–48 h. In contrast, cells exposed to the niosomal combination showed a persistent wound gap, indicating impaired migratory capability. This suggests that beyond killing cells, formulation also suppresses the cells’ motility.

Inhibition of migration has been noted with both components of therapy individually in other studies, CIS can inhibit migration and invasion in certain cancer cells by down-regulating pro-migratory pathway [39]. Moreover, combination treatments seem particularly effective: for example, a recent study showed that a natural Flavonoid (Apigenin) combined with VOR significantly inhibited wound closure in aggressive triple-negative breast cancer cells, thereby reducing their metastatic potential [36,40]. CIS–VOR combo appears to act similarly, if not more strongly, on colon, lung, and pancreatic cancer cells. The underlying mechanism might be multifaceted. By inducing robust apoptosis, fewer cells remain alive to migrate. CIS, when delivered effectively, might also trigger signalling changes that impede migration; for instance, it can suppress the Epithelial-to-Mesenchymal Transition (EMT) in some contexts. The net result was a clear attenuation of migration in the treated wounds. This outcome is very encouraging from a therapeutic standpoint: it implies that niosomal formulation could help not only in killing primary tumour cells but also in preventing the spread of those cells that survive. In the broader perspective, nanoparticle-mediated co-delivery systems are being explored not just for cytotoxic impact but also for anti-metastatic effect [36]. The current findings contribute to this narrative by demonstrating that a co-loaded niosomes can indeed impair a key step of the metastatic cascade (cell migration) in multiple cancer types. This is a valuable addition to the literature, as it extends the role of CIS–HDAC Inhibitor combinations to the realm of metastasis prevention, a topic that has begun gaining attention in recent years.

## 5. Conclusion

In the current study, the design, characterization, and in vitro evaluation of niosomal nanoparticles co-loaded with CIS and VOR were successfully demonstrated as a novel strategy for cancer therapy. The developed formulation utilized the ethanol injection method to encapsulate both a hydrophilic (CIS) and a hydrophobic (VOR) agent within a non-ionic surfactant-based vesicle system. The niosomes displayed favorable physicochemical characteristics, including nanoscale Particle Size, low Polydispersity Index, and moderate negative zeta potential, indicating colloidal stability and uniform distribution. The biological evaluation of the formulations revealed substantial enhancements in anticancer activity. The NIO-CIS-VOR formulation significantly decreased the IC₅₀ values in HT-29, A549, and Pnac-1 cancer cell lines compared to free drug treatments. Furthermore, it induced a pronounced increase in Caspase-3/7 activity, confirming apoptosis as the primary mechanism of cell death. The formulation also impaired cancer cell migration and completely abrogated clonogenic potential, demonstrating its multifaceted therapeutic benefits.

## Supporting information

S1 Fig FTIR Spectrum of Niosomes Nanoparticle NPs. (DOCX)

S2 Fig Intensity-based DLS size distribution profiles of Niosomes nanoparticle formulations. Representative DLS data displaying the hydrodynamic size distribution (by intensity) for NPs, is-NPs, VOR-NPs, and DLNPs. All formulations exhibited unimodal distributions with narrow peaks, indicating uniform particle populations and absence of aggregation.
(DOCX)

S3 Fig Combocyn report for all used cell lines Data represent the mean ± SD of three independent experiments. (DOCX)

S4 Fig HPLC representative raw data. (DOCX)
