# Supplementary material for: Development of niosomal nanoparticles loaded with cisplatin and vorinostat combination for cancer therapy
Source: PLoS One. 2026 Feb 6;21(2):e0342344. doi: 10.1371/journal.pone.0342344 (PMC12880632; doi:10.1371/journal.pone.0342344)
Supplement: S2 Fig — Representative DLS data displaying the hydrodynamic size distribution (by intensity) for NPs, is-NPs, VOR-NPs, and DLNPs. All formulations exhibited unimodal distributions with narrow peaks, indicating uniform particle populations and absence of aggregation. (DOCX) [file pone.0342344.s002.docx]

## **
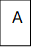
**


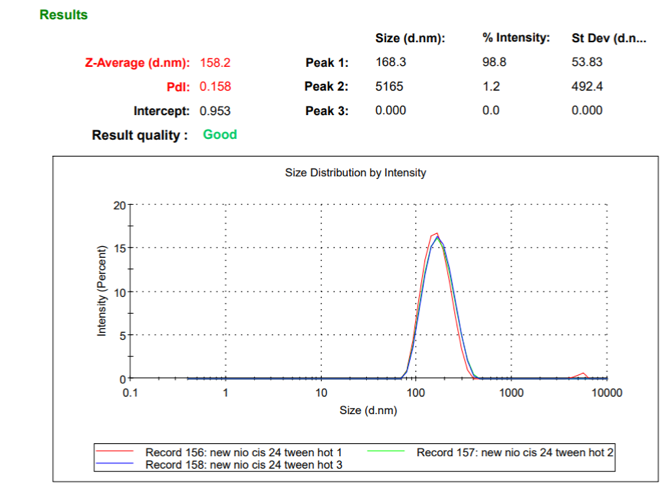

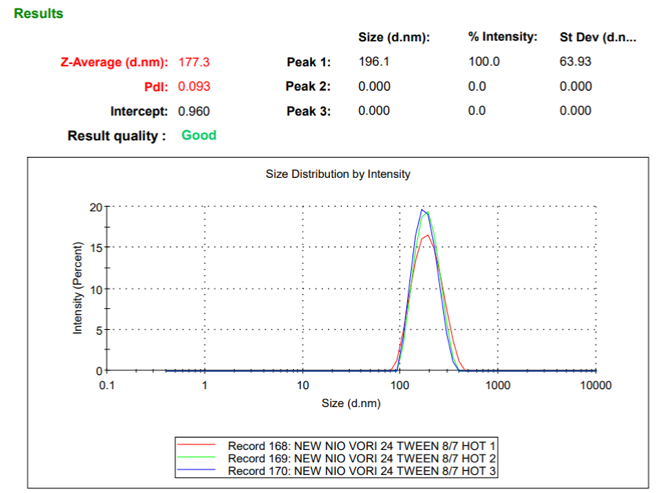

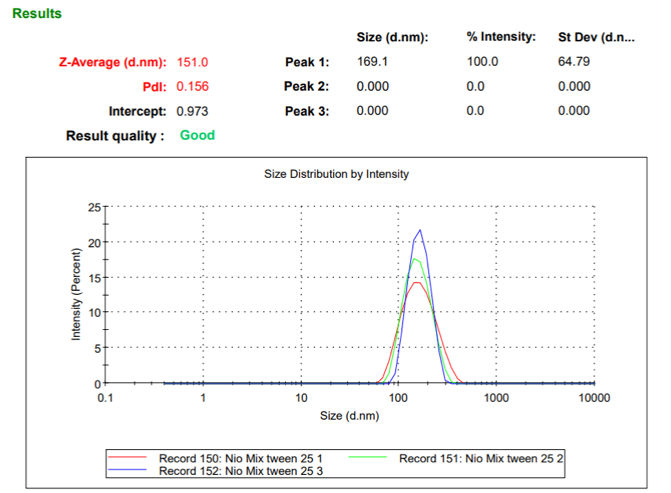

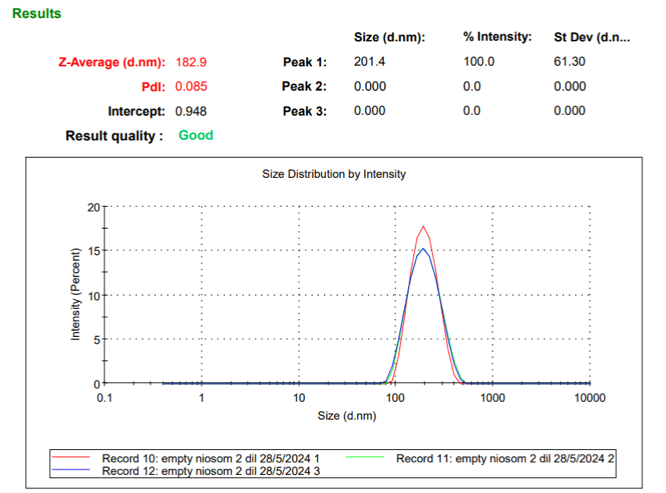


**NIO**

**NIO-CIS**

**NIO-VOR**

**NIO-CIS-VOR**


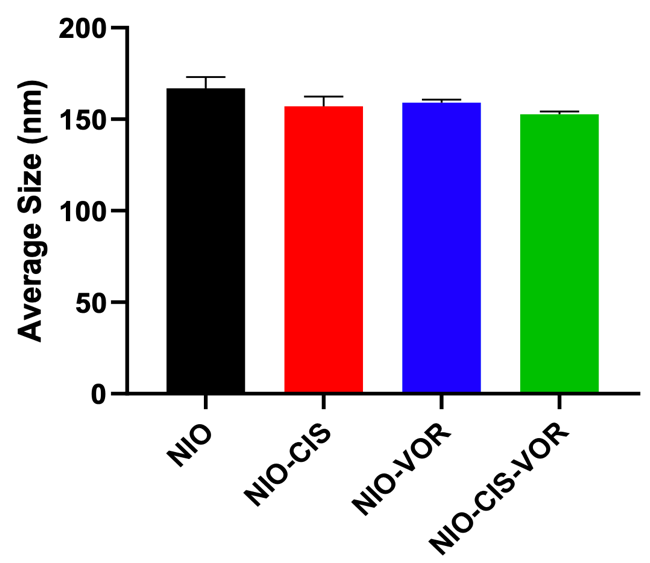

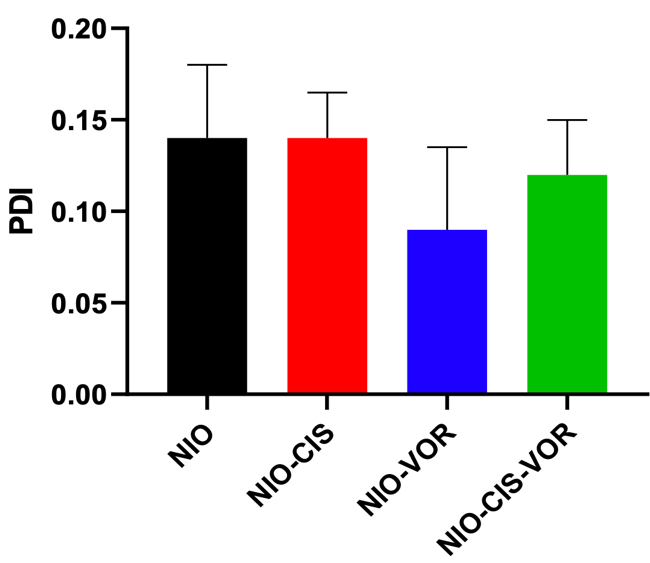


**Figure 24. Physical characterization of the niosomal nanoparticle formulations in terms of size (A) and PDI (B) with the a sample of the corresponding peaks.**
