# Supplementary material for: Development of niosomal nanoparticles loaded with cisplatin and vorinostat combination for cancer therapy
Source: PLoS One. 2026 Feb 6;21(2):e0342344. doi: 10.1371/journal.pone.0342344 (PMC12880632; doi:10.1371/journal.pone.0342344)
Supplement: S3 Fig — (DOCX) [file pone.0342344.s003.docx]

CompuSyn Report HT29

| **Experiment Name:** | NIO-CIS-VOR |
| --- | --- |
| **Date:** |  |
| **File Name:** | C:\Users\asha_\Desktop\CIS-VOR.cse |
| **Description** |  |

| **Drug:** | NIO-VOR (VOR) [Micrmol] |
| --- | --- |
| **Drug:** | Nio-CIS (CIS) [Micromolar] |
| **Drug Combo:** | NIO-CIS-VOR (C-V) (VOR+CIS [1:1]) |

Data for Drug: VOR [Micrmol]

| **Dose** | **Effect** |
| --- | --- |
| 50.0 | 0.09 |
| 25.0 | 0.15 |
| 12.5 | 0.17 |
| 6.25 | 0.22 |
| 3.125 | 0.43 |
| 1.5626 | 0.7 |
| 0.78125 | 0.82 |

7 data points entered.

| **X-int:** | 0.47284 |
| --- | --- |
| **Y-int:** | 0.43750 +/- 0.10481 |
| **m:** | -0.9253 +/- 0.10502 |
| **Dm:** | 2.97056 |
| **r:** | -0.9693 |

Data for Drug: CIS [Micromolar]

| **Dose** | **Effect** |
| --- | --- |
| 50.0 | 0.015 |
| 25.0 | 0.07 |
| 12.5 | 0.18 |
| 6.25 | 0.22 |
| 3.125 | 0.35 |
| 1.26525 | 0.82 |
| 0.78125 | 0.94 |

7 data points entered.

| **X-int:** | 0.53992 |
| --- | --- |
| **Y-int:** | 0.81438 +/- 0.13806 |
| **m:** | -1.5083 +/- 0.13861 |
| **Dm:** | 3.46674 |
| **r:** | -0.9795 |

Data for Drug Combo: C-V (VOR+CIS [1:1])

| **Dose A** | **Effect** |
| --- | --- |
| 25.0+ | 0.015 |
| 12.5+ | 0.04 |
| 6.25+ | 0.16 |
| 3.125+ | 0.23 |
| 1.5625+ | 0.34 |
| 0.78125+ | 0.57 |
| 0.391+ | 0.89 |

7 data points entered.

| **X-int:** | 0.41234 |
| --- | --- |
| **Y-int:** | 0.56820 +/- 0.10915 |
| **m:** | -1.3780 +/- 0.10938 |
| **Dm:** | 2.58430 |
| **r:** | -0.9846 |

Dose-Effect Curve

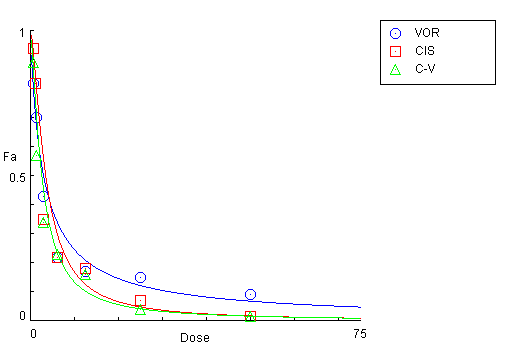


Median-Effect Plot

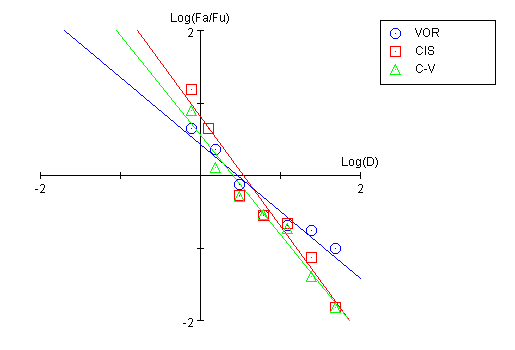


CI Data for Drug Combo: C-V (VOR+CIS [1:1])

| **Fa** | **CI Value** | **Total Dose** |
| --- | --- | --- |
| 0.05 | 0.60122 | 21.8943 |
| 0.1 | 0.62716 | 12.7301 |
| 0.15 | 0.65052 | 9.09976 |
| 0.2 | 0.67247 | 7.06733 |
| 0.25 | 0.69380 | 5.73572 |
| 0.3 | 0.71504 | 4.77949 |
| 0.35 | 0.73663 | 4.04988 |
| 0.4 | 0.75899 | 3.46842 |
| 0.45 | 0.78252 | 2.98942 |
| 0.5 | 0.80771 | 2.58430 |
| 0.55 | 0.83517 | 2.23407 |
| 0.6 | 0.86571 | 1.92554 |
| 0.65 | 0.90045 | 1.64908 |
| 0.7 | 0.94111 | 1.39734 |
| 0.75 | 0.99043 | 1.16438 |
| 0.8 | 1.05331 | 0.94499 |
| 0.85 | 1.13962 | 0.73393 |
| 0.9 | 1.27383 | 0.52463 |
| 0.95 | 1.54733 | 0.30504 |
| 0.97 | 1.79428 | 0.20739 |

CI values for actual experimental points:

| **Total Dose** | **Fa** | **CI Value** |
| --- | --- | --- |
| 50.0 | 0.015 | 0.54134 |
| 25.0 | 0.04 | 0.57410 |
| 12.5 | 0.16 | 0.95101 |
| 6.25 | 0.23 | 0.68961 |
| 3.125 | 0.34 | 0.54718 |
| 1.5625 | 0.57 | 0.62831 |
| 0.782 | 0.89 | 1.71196 |

Combination Index Plot

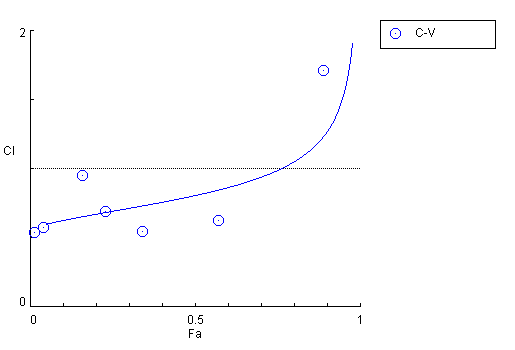


DRI Data for Drug Combo: C-V (VOR+CIS [1:1])

| **Fa** | **Dose VOR** | **Dose CIS** | **DRI VOR** | **DRI CIS** |
| --- | --- | --- | --- | --- |
| 0.05 | 71.5948 | 24.4181 | 6.54005 | 2.23055 |
| 0.1 | 31.9270 | 14.8788 | 5.01597 | 2.33756 |
| 0.15 | 19.3649 | 10.9487 | 4.25613 | 2.40638 |
| 0.2 | 13.2901 | 8.69114 | 3.76100 | 2.45952 |
| 0.25 | 9.73864 | 7.18198 | 3.39579 | 2.50430 |
| 0.3 | 7.42229 | 6.07972 | 3.10589 | 2.54409 |
| 0.35 | 5.79962 | 5.22590 | 2.86409 | 2.58077 |
| 0.4 | 4.60419 | 4.53594 | 2.65492 | 2.61557 |
| 0.45 | 3.69001 | 3.96005 | 2.46872 | 2.64938 |
| 0.5 | 2.97056 | 3.46674 | 2.29893 | 2.68293 |
| 0.55 | 2.39138 | 3.03489 | 2.14082 | 2.71691 |
| 0.6 | 1.91656 | 2.64958 | 1.99067 | 2.75203 |
| 0.65 | 1.52152 | 2.29976 | 1.84529 | 2.78914 |
| 0.7 | 1.18888 | 1.97679 | 1.70163 | 2.82935 |
| 0.75 | 0.90610 | 1.67340 | 1.55637 | 2.87430 |
| 0.8 | 0.66397 | 1.38282 | 1.40523 | 2.92663 |
| 0.85 | 0.45568 | 1.09769 | 1.24176 | 2.99126 |
| 0.9 | 0.27639 | 0.80775 | 1.05365 | 3.07932 |
| 0.95 | 0.12325 | 0.49219 | 0.80811 | 3.22706 |
| 0.97 | 0.06938 | 0.34598 | 0.66909 | 3.33648 |

DRI values calculated at experimental points

| **Fa** | **Dose VOR** | **Dose CIS** | **DRI VOR** | **DRI CIS** |
| --- | --- | --- | --- | --- |
| 0.015 | 273.512 | 55.5634 | 10.9405 | 2.22253 |
| 0.04 | 92.1583 | 28.5087 | 7.37266 | 2.28069 |
| 0.16 | 17.8307 | 10.4082 | 2.85291 | 1.66531 |
| 0.23 | 10.9645 | 7.72378 | 3.50864 | 2.47161 |
| 0.34 | 6.08375 | 5.38150 | 3.89360 | 3.44416 |
| 0.57 | 2.19051 | 2.87586 | 2.80385 | 3.68111 |
| 0.89 | 0.31010 | 0.86683 | 0.79309 | 2.21697 |

DRI Plot for Combo: C-V (VOR+CIS [1:1])

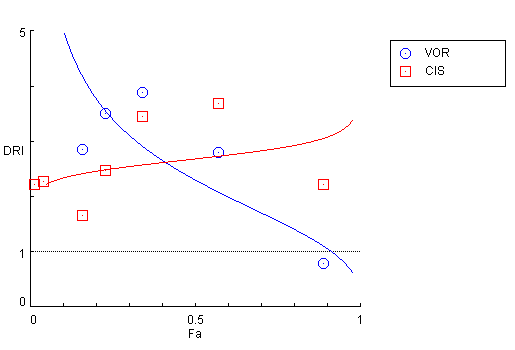


Isobologram for Combo: C-V (VOR+CIS [1:1])

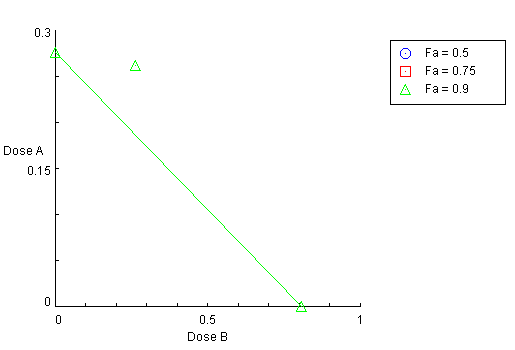


Summary Table

| **Experiment Name:** | NIO-CIS-VOR |
| --- | --- |
| **Date:** |  |
| **File Name:** | C:\Users\asha_\Desktop\CIS-VOR.cse |
| **Description** |  |

| **Drug:** | NIO-VOR (VOR) [Micrmol] |
| --- | --- |
| **Drug:** | Nio-CIS (CIS) [Micromolar] |
| **Drug Combo:** | NIO-CIS-VOR (C-V) (VOR+CIS [1:1]) |

| **Drug/Combo** | **Dm** | **m** | **r** |
| --- | --- | --- | --- |
| VOR | 2.97056 | -0.9253 | -0.9693 |
| CIS | 3.46674 | -1.5083 | -0.9795 |
| C-V | 2.58430 | -1.3780 | -0.9846 |

|  | CI values at: | | | |
| --- | --- | --- | --- | --- |
| **Combo** | **ED50** | **ED75** | **ED90** | **ED95** |
| C-V | 0.80771 | 0.99043 | 1.27383 | 1.54733 |

Data for Fa = 0.5

| **Drug/Combo** | **CI value** | **Dose VOR** | **Dose CIS** |
| --- | --- | --- | --- |
| VOR |  | 2.97056 |  |
| CIS |  |  | 3.46674 |
| C-V | 0.80771 | 1.29215 | 1.29215 |

Data for Fa = 0.75

| **Drug/Combo** | **CI value** | **Dose VOR** | **Dose CIS** |
| --- | --- | --- | --- |
| VOR |  | 0.90610 |  |
| CIS |  |  | 1.67340 |
| C-V | 0.99043 | 0.58219 | 0.58219 |

Data for Fa = 0.9

| **Drug/Combo** | **CI value** | **Dose VOR** | **Dose CIS** |
| --- | --- | --- | --- |
| VOR |  | 0.27639 |  |
| CIS |  |  | 0.80775 |
| C-V | 1.27383 | 0.26231 | 0.26231 |

Data for Fa = 0.95

| **Drug/Combo** | **CI value** | **Dose VOR** | **Dose CIS** |
| --- | --- | --- | --- |
| VOR |  | 0.12325 |  |
| CIS |  |  | 0.49219 |
| C-V | 1.54733 | 0.15252 | 0.15252 |

Data for Fa = 0.97

| **Drug/Combo** | **CI value** | **Dose VOR** | **Dose CIS** |
| --- | --- | --- | --- |
| VOR |  | 0.06938 |  |
| CIS |  |  | 0.34598 |
| C-V | 1.79428 | 0.10370 | 0.10370 |

CompuSyn Report

| **Experiment Name:** | NIO-CIS-VOR |
| --- | --- |
| **Date:** |  |
| **File Name:** | C:\Users\asha_\Desktop\CIS-VOR.cse |
| **Description** | A549 |

| **Drug:** | NIO-VOR (VOR) [Micrmol] |
| --- | --- |
| **Drug:** | Nio-CIS (CIS) [Micromolar] |
| **Drug Combo:** | NIO-CIS-VOR (C-V) (VOR+CIS [1:1]) |

Data for Drug: VOR [Micrmol]

| **Dose** | **Effect** |
| --- | --- |
| 50.0 | 0.09 |
| 25.0 | 0.1 |
| 12.5 | 0.13 |
| 6.25 | 0.15 |
| 3.125 | 0.25 |
| 1.5626 | 0.43 |
| 0.78125 | 0.65 |

7 data points entered.

| **X-int:** | -0.0027 |
| --- | --- |
| **Y-int:** | -0.0019 +/- 0.10140 |
| **m:** | -0.6920 +/- 0.10161 |
| **Dm:** | 0.99377 |
| **r:** | -0.9501 |

Data for Drug: CIS [Micromolar]

| **Dose** | **Effect** |
| --- | --- |
| 50.0 | 0.07 |
| 25.0 | 0.09 |
| 12.5 | 0.15 |
| 6.25 | 0.24 |
| 3.125 | 0.42 |
| 1.26525 | 0.53 |
| 0.78125 | 0.75 |

7 data points entered.

| **X-int:** | 0.29145 |
| --- | --- |
| **Y-int:** | 0.25362 +/- 0.06086 |
| **m:** | -0.8702 +/- 0.06111 |
| **Dm:** | 1.95635 |
| **r:** | -0.9879 |

Data for Drug Combo: C-V (VOR+CIS [1:1])

| **Dose A** | **Effect** |
| --- | --- |
| 25.0+ | 0.08 |
| 12.5+ | 0.1 |
| 6.25+ | 0.13 |
| 3.125+ | 0.17 |
| 1.5625+ | 0.24 |
| 0.78125+ | 0.42 |
| 0.391+ | 0.56 |

7 data points entered.

| **X-int:** | -0.1022 |
| --- | --- |
| **Y-int:** | -0.0661 +/- 0.06348 |
| **m:** | -0.6466 +/- 0.06361 |
| **Dm:** | 0.79030 |
| **r:** | -0.9766 |

Dose-Effect Curve

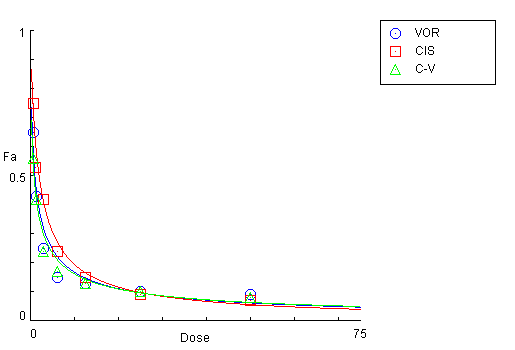


Median-Effect Plot

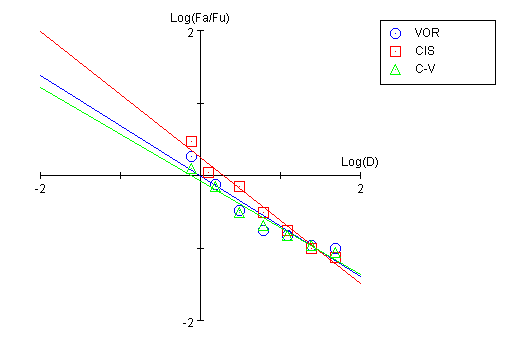


CI Data for Drug Combo: C-V (VOR+CIS [1:1])

| **Fa** | **CI Value** | **Total Dose** |
| --- | --- | --- |
| 0.05 | 1.18709 | 75.0680 |
| 0.1 | 0.98068 | 23.6363 |
| 0.15 | 0.87665 | 11.5572 |
| 0.2 | 0.80815 | 6.74388 |
| 0.25 | 0.75711 | 4.32200 |
| 0.3 | 0.71620 | 2.93013 |
| 0.35 | 0.68174 | 2.05861 |
| 0.4 | 0.65164 | 1.47953 |
| 0.45 | 0.62456 | 1.07788 |
| 0.5 | 0.59961 | 0.79030 |
| 0.55 | 0.57610 | 0.57944 |
| 0.6 | 0.55351 | 0.42214 |
| 0.65 | 0.53133 | 0.30339 |
| 0.7 | 0.50908 | 0.21315 |
| 0.75 | 0.48617 | 0.14451 |
| 0.8 | 0.46183 | 0.09261 |
| 0.85 | 0.43478 | 0.05404 |
| 0.9 | 0.40245 | 0.02642 |
| 0.95 | 0.35754 | 0.00832 |
| 0.97 | 0.33010 | 0.00366 |

CI values for actual experimental points:

| **Total Dose** | **Fa** | **CI Value** |
| --- | --- | --- |
| 50.0 | 0.08 | 1.50977 |
| 25.0 | 0.1 | 1.03726 |
| 12.5 | 0.13 | 0.76283 |
| 6.25 | 0.17 | 0.57631 |
| 3.125 | 0.24 | 0.50965 |
| 1.5625 | 0.42 | 0.76870 |
| 0.782 | 0.56 | 0.82117 |

Combination Index Plot

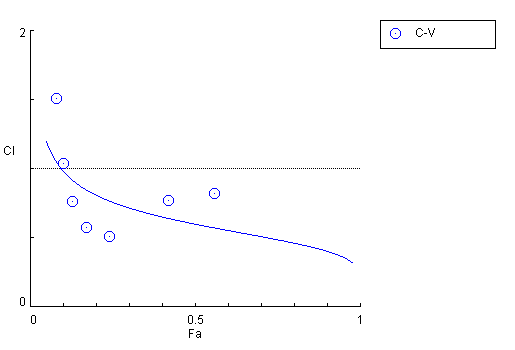


DRI Data for Drug Combo: C-V (VOR+CIS [1:1])

| **Fa** | **Dose VOR** | **Dose CIS** | **DRI VOR** | **DRI CIS** |
| --- | --- | --- | --- | --- |
| 0.05 | 69.9986 | 57.6664 | 1.86494 | 1.53638 |
| 0.1 | 23.7777 | 24.4350 | 2.01196 | 2.06758 |
| 0.15 | 12.1856 | 14.3592 | 2.10874 | 2.48489 |
| 0.2 | 7.36657 | 9.62282 | 2.18467 | 2.85379 |
| 0.25 | 4.86104 | 6.91400 | 2.24944 | 3.19944 |
| 0.3 | 3.38076 | 5.17972 | 2.30759 | 3.53549 |
| 0.35 | 2.43091 | 3.98464 | 2.36170 | 3.87120 |
| 0.4 | 1.78542 | 3.11746 | 2.41349 | 4.21412 |
| 0.45 | 1.32807 | 2.46374 | 2.46421 | 4.57144 |
| 0.5 | 0.99377 | 1.95635 | 2.51494 | 4.95093 |
| 0.55 | 0.74363 | 1.55345 | 2.56672 | 5.36193 |
| 0.6 | 0.55314 | 1.22770 | 2.62066 | 5.81657 |
| 0.65 | 0.40626 | 0.96051 | 2.67812 | 6.33182 |
| 0.7 | 0.29212 | 0.73890 | 2.74093 | 6.93305 |
| 0.75 | 0.20316 | 0.55356 | 2.81178 | 7.66125 |
| 0.8 | 0.13406 | 0.39773 | 2.89514 | 8.58917 |
| 0.85 | 0.08105 | 0.26654 | 2.99938 | 9.86431 |
| 0.9 | 0.04153 | 0.15663 | 3.14366 | 11.8553 |
| 0.95 | 0.01411 | 0.06637 | 3.39149 | 15.9542 |
| 0.97 | 0.00654 | 0.03603 | 3.57964 | 19.7077 |

DRI values calculated at experimental points

| **Fa** | **Dose VOR** | **Dose CIS** | **DRI VOR** | **DRI CIS** |
| --- | --- | --- | --- | --- |
| 0.08 | 33.8843 | 32.3851 | 1.35537 | 1.29540 |
| 0.1 | 23.7777 | 24.4350 | 1.90222 | 1.95480 |
| 0.13 | 15.4970 | 17.3842 | 2.47951 | 2.78147 |
| 0.17 | 9.82558 | 12.0999 | 3.14419 | 3.87198 |
| 0.24 | 5.25604 | 7.35720 | 3.36387 | 4.70861 |
| 0.42 | 1.58433 | 2.83487 | 2.02794 | 3.62863 |
| 0.56 | 0.70137 | 1.48283 | 1.79377 | 3.79240 |

DRI Plot for Combo: C-V (VOR+CIS [1:1])

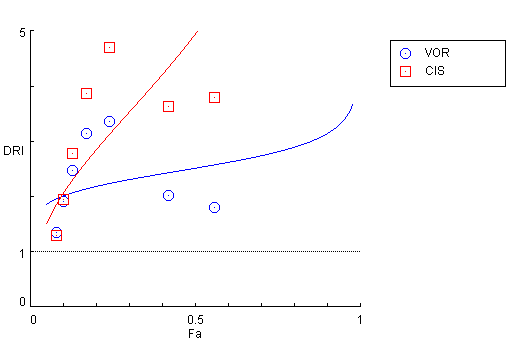


Isobologram for Combo: C-V (VOR+CIS [1:1])

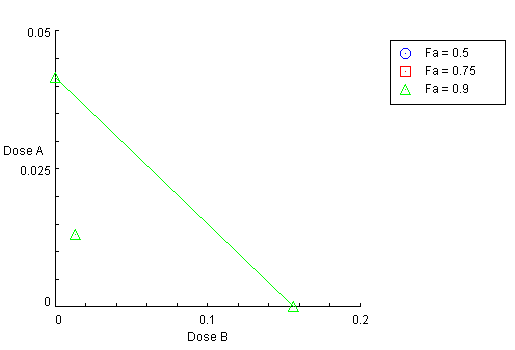


Summary Table

| **Experiment Name:** | NIO-CIS-VOR |
| --- | --- |
| **Date:** |  |
| **File Name:** | C:\Users\asha_\Desktop\CIS-VOR.cse |
| **Description** | A549 |

| **Drug:** | NIO-VOR (VOR) [Micrmol] |
| --- | --- |
| **Drug:** | Nio-CIS (CIS) [Micromolar] |
| **Drug Combo:** | NIO-CIS-VOR (C-V) (VOR+CIS [1:1]) |

| **Drug/Combo** | **Dm** | **m** | **r** |
| --- | --- | --- | --- |
| VOR | 0.99377 | -0.6920 | -0.9501 |
| CIS | 1.95635 | -0.8702 | -0.9879 |
| C-V | 0.79030 | -0.6466 | -0.9766 |

|  | CI values at: | | | |
| --- | --- | --- | --- | --- |
| **Combo** | **ED50** | **ED75** | **ED90** | **ED95** |
| C-V | 0.59961 | 0.48617 | 0.40245 | 0.35754 |

Data for Fa = 0.5

| **Drug/Combo** | **CI value** | **Dose VOR** | **Dose CIS** |
| --- | --- | --- | --- |
| VOR |  | 0.99377 |  |
| CIS |  |  | 1.95635 |
| C-V | 0.59961 | 0.39515 | 0.39515 |

Data for Fa = 0.75

| **Drug/Combo** | **CI value** | **Dose VOR** | **Dose CIS** |
| --- | --- | --- | --- |
| VOR |  | 0.20316 |  |
| CIS |  |  | 0.55356 |
| C-V | 0.48617 | 0.07225 | 0.07225 |

Data for Fa = 0.9

| **Drug/Combo** | **CI value** | **Dose VOR** | **Dose CIS** |
| --- | --- | --- | --- |
| VOR |  | 0.04153 |  |
| CIS |  |  | 0.15663 |
| C-V | 0.40245 | 0.01321 | 0.01321 |

Data for Fa = 0.95

| **Drug/Combo** | **CI value** | **Dose VOR** | **Dose CIS** |
| --- | --- | --- | --- |
| VOR |  | 0.01411 |  |
| CIS |  |  | 0.06637 |
| C-V | 0.35754 | 0.00416 | 0.00416 |

Data for Fa = 0.97

| **Drug/Combo** | **CI value** | **Dose VOR** | **Dose CIS** |
| --- | --- | --- | --- |
| VOR |  | 0.00654 |  |
| CIS |  |  | 0.03603 |
| C-V | 0.33010 | 0.00183 | 0.00183 |

CompuSyn Report

| **Experiment Name:** | NIO-CIS-VOR |
| --- | --- |
| **Date:** |  |
| **File Name:** | C:\Users\asha_\Desktop\CIS-VOR.cse |
| **Description** | PANC 1 |

| **Drug:** | NIO-VOR (VOR) [Micrmol] |
| --- | --- |
| **Drug:** | Nio-CIS (CIS) [Micromolar] |
| **Drug Combo:** | NIO-CIS-VOR (C-V) (VOR+CIS [1:1]) |

Data for Drug: VOR [Micrmol]

| **Dose** | **Effect** |
| --- | --- |
| 50.0 | 0.27 |
| 25.0 | 0.34 |
| 12.5 | 0.4 |
| 6.25 | 0.75 |
| 3.125 | 0.95 |
| 1.5626 | 0.98 |
| 0.78125 | 0.99 |

7 data points entered.

| **X-int:** | 1.22706 |
| --- | --- |
| **Y-int:** | 1.84801 +/- 0.15242 |
| **m:** | -1.5060 +/- 0.15273 |
| **Dm:** | 16.8678 |
| **r:** | -0.9752 |

Data for Drug: CIS [Micromolar]

| **Dose** | **Effect** |
| --- | --- |
| 50.0 | 0.25 |
| 25.0 | 0.36 |
| 12.5 | 0.42 |
| 6.25 | 0.53 |
| 3.125 | 0.75 |
| 1.26525 | 0.98 |
| 0.78125 | 0.99 |

7 data points entered.

| **X-int:** | 1.12615 |
| --- | --- |
| **Y-int:** | 1.56864 +/- 0.21647 |
| **m:** | -1.3929 +/- 0.21734 |
| **Dm:** | 13.3706 |
| **r:** | -0.9442 |

Data for Drug Combo: C-V (VOR+CIS [1:1])

| **Dose A** | **Effect** |
| --- | --- |
| 25.0+ | 0.35 |
| 12.5+ | 0.4 |
| 6.25+ | 0.47 |
| 3.125+ | 0.55 |
| 1.5625+ | 0.87 |
| 0.78125+ | 0.9 |
| 0.391+ | 0.99 |

7 data points entered.

| **X-int:** | 1.20393 |
| --- | --- |
| **Y-int:** | 1.41874 +/- 0.20739 |
| **m:** | -1.1784 +/- 0.20782 |
| **Dm:** | 15.9930 |
| **r:** | -0.9303 |

Dose-Effect Curve

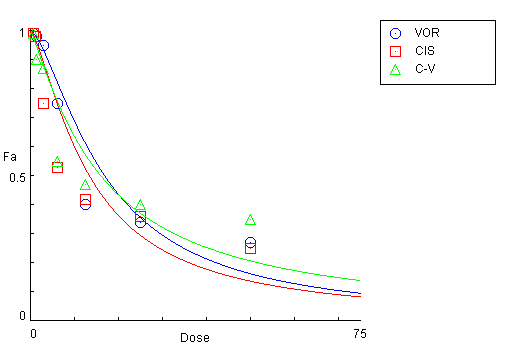


Median-Effect Plot

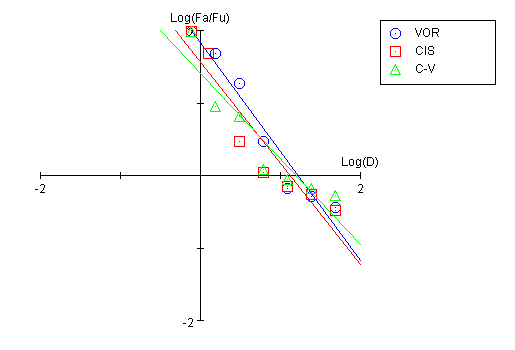


CI Data for Drug Combo: C-V (VOR+CIS [1:1])

| **Fa** | **CI Value** | **Total Dose** |
| --- | --- | --- |
| 0.05 | 1.69513 | 194.569 |
| 0.1 | 1.50819 | 103.204 |
| 0.15 | 1.40322 | 69.6947 |
| 0.2 | 1.32917 | 51.8603 |
| 0.25 | 1.27105 | 40.6268 |
| 0.3 | 1.22242 | 32.8241 |
| 0.35 | 1.17992 | 27.0440 |
| 0.4 | 1.14152 | 22.5611 |
| 0.45 | 1.10592 | 18.9620 |
| 0.5 | 1.07213 | 15.9930 |
| 0.55 | 1.03941 | 13.4889 |
| 0.6 | 1.00708 | 11.3371 |
| 0.65 | 0.97446 | 9.45779 |
| 0.7 | 0.94080 | 7.79233 |
| 0.75 | 0.90513 | 6.29576 |
| 0.8 | 0.86599 | 4.93203 |
| 0.85 | 0.82093 | 3.66996 |
| 0.9 | 0.76479 | 2.47837 |
| 0.95 | 0.68233 | 1.31458 |
| 0.97 | 0.62927 | 0.83724 |

CI values for actual experimental points:

| **Total Dose** | **Fa** | **CI Value** |
| --- | --- | --- |
| 50.0 | 0.35 | 2.18148 |
| 25.0 | 0.4 | 1.26493 |
| 12.5 | 0.47 | 0.77093 |
| 6.25 | 0.55 | 0.48161 |
| 3.125 | 0.87 | 0.78476 |
| 1.5625 | 0.9 | 0.48217 |
| 0.782 | 0.99 | 1.28199 |

Combination Index Plot

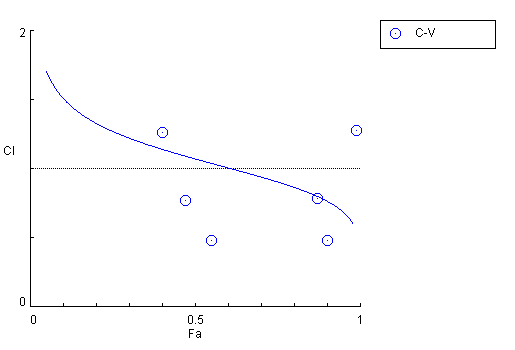


DRI Data for Drug Combo: C-V (VOR+CIS [1:1])

| **Fa** | **Dose VOR** | **Dose CIS** | **DRI VOR** | **DRI CIS** |
| --- | --- | --- | --- | --- |
| 0.05 | 119.162 | 110.710 | 1.22489 | 1.13800 |
| 0.1 | 72.5550 | 64.7463 | 1.40606 | 1.25473 |
| 0.15 | 53.3656 | 46.4489 | 1.53141 | 1.33293 |
| 0.2 | 42.3469 | 36.1725 | 1.63312 | 1.39500 |
| 0.25 | 34.9835 | 29.4227 | 1.72219 | 1.44844 |
| 0.3 | 29.6069 | 24.5656 | 1.80397 | 1.49680 |
| 0.35 | 25.4431 | 20.8525 | 1.88161 | 1.54212 |
| 0.4 | 22.0791 | 17.8883 | 1.95728 | 1.58577 |
| 0.45 | 19.2720 | 15.4425 | 2.03269 | 1.62878 |
| 0.5 | 16.8678 | 13.3706 | 2.10940 | 1.67206 |
| 0.55 | 14.7636 | 11.5767 | 2.18901 | 1.71648 |
| 0.6 | 12.8865 | 9.99385 | 2.27335 | 1.76304 |
| 0.65 | 11.1828 | 8.57321 | 2.36477 | 1.81294 |
| 0.7 | 9.61007 | 7.27739 | 2.46655 | 1.86783 |
| 0.75 | 8.13309 | 6.07602 | 2.58367 | 1.93020 |
| 0.8 | 6.71889 | 4.94224 | 2.72459 | 2.00414 |
| 0.85 | 5.33160 | 3.84881 | 2.90554 | 2.09747 |
| 0.9 | 3.92150 | 2.76113 | 3.16458 | 2.22819 |
| 0.95 | 2.38770 | 1.61479 | 3.63264 | 2.45674 |
| 0.97 | 1.67751 | 1.10243 | 4.00725 | 2.63350 |

DRI values calculated at experimental points

| **Fa** | **Dose VOR** | **Dose CIS** | **DRI VOR** | **DRI CIS** |
| --- | --- | --- | --- | --- |
| 0.35 | 25.4431 | 20.8525 | 1.01772 | 0.83410 |
| 0.4 | 22.0791 | 17.8883 | 1.76633 | 1.43107 |
| 0.47 | 18.2686 | 14.5751 | 2.92298 | 2.33201 |
| 0.55 | 14.7636 | 11.5767 | 4.72436 | 3.70454 |
| 0.87 | 4.77403 | 3.41554 | 3.05538 | 2.18594 |
| 0.9 | 3.92150 | 2.76113 | 5.01951 | 3.53425 |
| 0.99 | 0.79795 | 0.49369 | 2.04080 | 1.26264 |

DRI Plot for Combo: C-V (VOR+CIS [1:1])

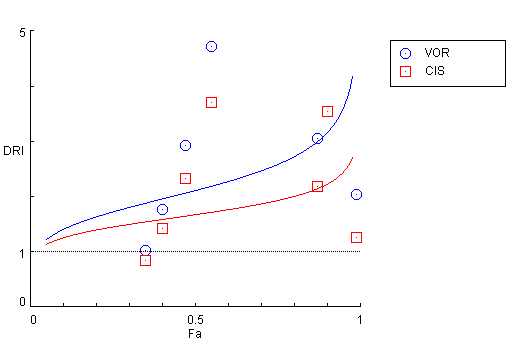


Isobologram for Combo: C-V (VOR+CIS [1:1])

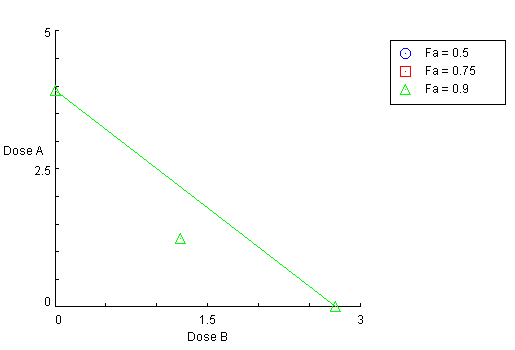


Summary Table

| **Experiment Name:** | NIO-CIS-VOR |
| --- | --- |
| **Date:** |  |
| **File Name:** | C:\Users\asha_\Desktop\CIS-VOR.cse |
| **Description** | PANC 1 |

| **Drug:** | NIO-VOR (VOR) [Micrmol] |
| --- | --- |
| **Drug:** | Nio-CIS (CIS) [Micromolar] |
| **Drug Combo:** | NIO-CIS-VOR (C-V) (VOR+CIS [1:1]) |

| **Drug/Combo** | **Dm** | **m** | **r** |
| --- | --- | --- | --- |
| VOR | 16.8678 | -1.5060 | -0.9752 |
| CIS | 13.3706 | -1.3929 | -0.9442 |
| C-V | 15.9930 | -1.1784 | -0.9303 |

|  | CI values at: | | | |
| --- | --- | --- | --- | --- |
| **Combo** | **ED50** | **ED75** | **ED90** | **ED95** |
| C-V | 1.07213 | 0.90513 | 0.76479 | 0.68233 |

Data for Fa = 0.5

| **Drug/Combo** | **CI value** | **Dose VOR** | **Dose CIS** |
| --- | --- | --- | --- |
| VOR |  | 16.8678 |  |
| CIS |  |  | 13.3706 |
| C-V | 1.07213 | 7.99650 | 7.99650 |

Data for Fa = 0.75

| **Drug/Combo** | **CI value** | **Dose VOR** | **Dose CIS** |
| --- | --- | --- | --- |
| VOR |  | 8.13309 |  |
| CIS |  |  | 6.07602 |
| C-V | 0.90513 | 3.14788 | 3.14788 |

Data for Fa = 0.9

| **Drug/Combo** | **CI value** | **Dose VOR** | **Dose CIS** |
| --- | --- | --- | --- |
| VOR |  | 3.92150 |  |
| CIS |  |  | 2.76113 |
| C-V | 0.76479 | 1.23918 | 1.23918 |

Data for Fa = 0.95

| **Drug/Combo** | **CI value** | **Dose VOR** | **Dose CIS** |
| --- | --- | --- | --- |
| VOR |  | 2.38770 |  |
| CIS |  |  | 1.61479 |
| C-V | 0.68233 | 0.65729 | 0.65729 |

Data for Fa = 0.97

| **Drug/Combo** | | **CI value** | **Dose VOR** | | **Dose CIS** | | |
| --- | --- | --- | --- | --- | --- | --- | --- |
| VOR | |  | 1.67751 | |  | | |
| CIS | |  |  | | 1.10243 | | |
| C-V | | 0.62927 | 0.41862 | | 0.41862 | | |
|  | |  |  | |  | | |
|  | |  |  | |  | | |
| Type | | CI @ Fa ≈ 0.5 | CI @ Fa ≈ 0.75 | | CI @ Fa ≈ 0.9 | | |
| HCT29 | | ~0.65 | ~0.80 | | ~1.10 | | |
| A549 | | ~0.80 | ~0.95 | | ~1.20 | | |
| PANC-1 | | ~0.90 | ~1.05 | | ~1.30 | | |
| **(Vorinostat)** |  |  |  |  |  |  |  |
| **HCT29** | NIO-CIS-VOR | | < 1 (Synergistic) | | ~2–3 | ~1.5–2 | |
| **A549** | NIO-CIS-VOR | | < 1 (Synergistic) | | ~2 | ~1.5 | |
| **PANC-1** | NIO-CIS-VOR | | < 1 (Synergistic) | | ~1.5 | ~1.2 | |
