# Supplementary figures and images for: Development of niosomal nanoparticles loaded with cisplatin and vorinostat combination for cancer therapy
Source: PLoS One. 2026 Feb 6;21(2):e0342344. doi: 10.1371/journal.pone.0342344 (PMC12880632; doi:10.1371/journal.pone.0342344)

S4 Fig. Hplc figures and validation

A


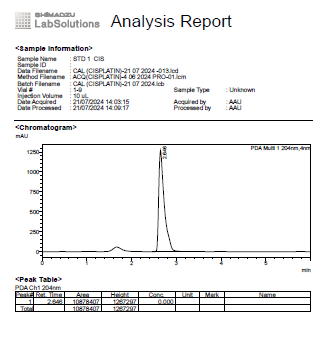


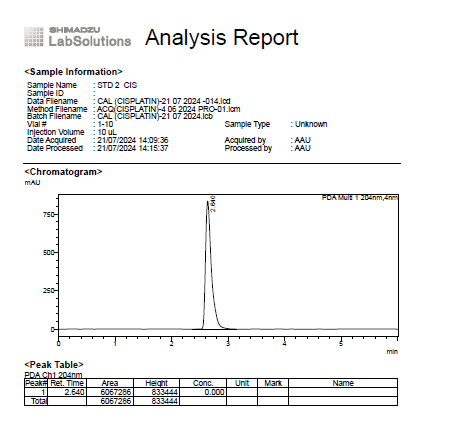


B


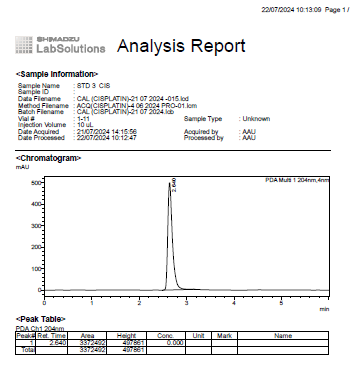

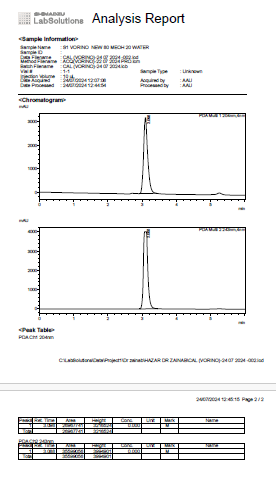


D

C

Supplement: S4 Fig — (DOCX) [file pone.0342344.s004.docx]
