# Supplementary material for: Development of niosomal nanoparticles loaded with cisplatin and vorinostat combination for cancer therapy
Source: PLoS One. 2026 Feb 6;21(2):e0342344. doi: 10.1371/journal.pone.0342344 (PMC12880632; doi:10.1371/journal.pone.0342344)
Supplement: Supplementary file 4 [file pone.0342344.s001.docx]

SHIMADZU

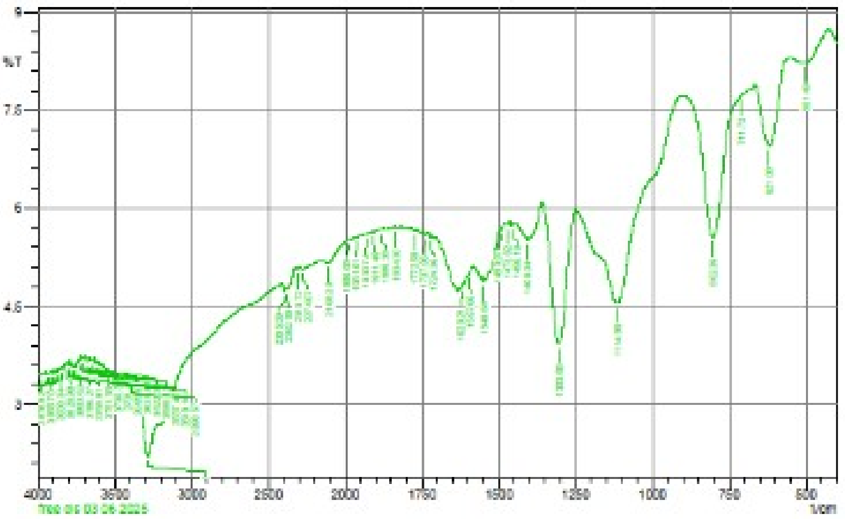

No.	Peak	Intensity	Corr. Intensity	Base (H)	Base (L)	Area	Corr. Area
1	501.49	8.2269	0.0157	503.42	430.13	78.6525	0.151
2	621.08	6.9506	1.0661	663.51	551.64	125.3916	3.2696
3	711.73	7.7095	0.0182	715.59	665.44	55.5815	0.036
4	802.39	5.5591	2.1582	902.69	715.59	217.7177	9.5702
5	1114.86	4.5435	2.1074	1249.87	904.61	425.1963	21.935
6	1303.88	3.93	2.0946	1367.89	1251.8	138.9464	9.4129
7	1408.04	5.5044	0.2186	1421.54	1369.82	76.8648	0.8237
8	1458.18	5.7333	0.0529	1463.97	1454.33	11.9546	0.0204
9	1473.62	5.7474	0.0241	1475.54	1465.9	11.9453	0.0107
10	1490.97	5.6604	0.0219	1492.9	1483.26	11.9903	0.0023
11	1548.84	4.8823	0.165	1552.7	1496.76	71.8565	0.4983
12	1597.06	4.9594	0.0245	1598.99	1587.42	14.997	0
13	1620.21	4.7956	0.0159	1622.13	1602.85	25.2858	0.0058
14	1724.36	5.5526	0.0491	1730.15	1718.58	14.8116	0.022
15	1737.86	5.5923	0.0331	1745.58	1732.08	16.8966	0.0216
16	1772.58	5.6514	0.0359	1780.3	1764.87	19.3436	0.0307
17	1834.3	5.7032	0.0157	1838.16	1828.52	11.9922	0.006
18	1886.38	5.6723	0.0178	1890.24	1876.74	16.818	0.0119
19	1511.46	5.6382	0.01	1913.39	1892.17	26.4729	0.0167
20	1500.74	5.6042	0.0051	1932.67	1916.31	21.6597	0.0086
21	1961.61	5.5565	0.0063	1963.53	1936.53	33.8507	0.0124
22	1986.68	5.5161	0.005	1988.61	1965.46	29.0922	0.0082
23	2108.2	5.1679	0.0112	2112.06	1988.61	157.0264	0.0758
24	2274.07	5.0627	0.0676	2299.15	2162.2	176.6697	0.3112
25	2310.72	5.0612	0.0484	2324.22	2301.08	29.9511	0.0544
26	2362.09	4.7596	0.0237	2385.95	2326.15	78.3525	0.2215
27	2395.59	4.7228	0.0651	2414.88	2387.87	35.657	0.0676
28	3294.77	2.2045	1.2819	3373.5	2416.81	1363.1829	30.7405
29	3398.57	3.3304	0.0056	3402.43	3375.43	39.8558	0.0087
30	3518.16	3.4608	0.0206	3531.66	3510.45	30.9389	0.0148
31	3537.45	3.4919	0.0187	3554.81	3533.59	30.8511	0.017
32	3599.17	3.5869	0.0592	3612.67	3593.38	27.7822	0.0634
33	3622.32	3.6263	0.0664	3631.96	3614.6	24.9502	0.0901

SHIMADZU

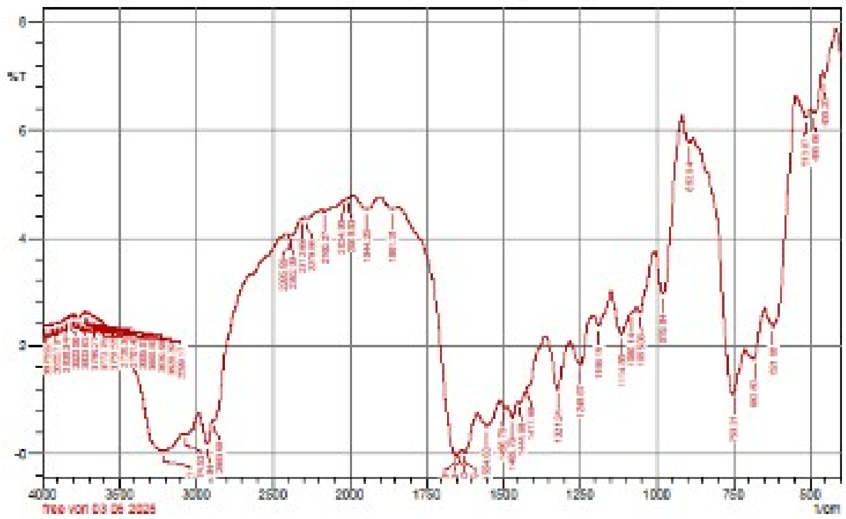

No.	Peak	Intensity	Corr. Intensity	Base (H)	Base (L)	Area	Corr. Area
1	455.2	6.9694	0.2241	460.99	416.62	50.2146	0.2784
2	486.06	6.3232	0.2609	496.71	462.92	36.8327	0.422
3	513.07	6.2304	0.2	545.85	503.42	50.6756	0.2765
4	621.08	2.3688	1.3222	646.15	547.78	143.5563	8.864
5	682.8	1.7964	0.1667	688.59	646.08	67.2168	0.4622
6	750.31	1.099	1.6617	881.47	715.59	252.2219	13.5798
7	893.04	5.769	0.2145	916.19	883.4	40.3088	0.3635
8	979.84	2.9693	1.5075	1004.91	918.12	120.4763	6.6137
9	1055.06	2.6188	0.2773	1064.71	1006.84	67.2249	0.9943
10	1080.14	2.6129	0.0313	1083.99	1066.64	27.3806	0.0943
11	1114.86	2.2141	0.5965	1147.65	1085.92	99.2031	3.4539
12	1188.15	2.3709	0.3326	1205.51	1149.57	88.7266	1.7633
13	1249.87	1.6888	0.6246	1280.73	1207.44	124.1466	4.263
14	1321.24	1.1989	0.9381	1361.74	1282.66	140.0260	7.8637
15	1417.68	1.2373	0.0376	1419.61	1363.67	98.2692	0.1582
16	1444.68	0.9441	0.0684	1452.4	1421.54	61.111	0.6646
17	1469.76	0.673	0.254	1489.05	1454.33	72.9674	2.3307
18	1496.76	0.8536	0.0662	1510.26	1490.97	39.4646	0.2399
19	1554.63	0.6043	0.332	1583.56	1512.19	165.8266	8.2005
20	1625.99	0.0353	0.1436	1633.71	1585.49	129.7474	6.2637
21	1641.42	0.0252	0.0111	1643.35	1635.64	24.804	0
22	1645.28	0	0.0085	1647.21	1645.28	7.8458	0
23	1658.78	0	-0.0031	1660.71	1656.85	771.5264	189.3542
24	1861.31	4.5335	0.0592	1903.74	1846.88	77.2763	0.25
25	1944.25	4.5343	0.2406	1982.82	1905.67	102.9159	0.9939
26	2009.83	4.7254	0.0039	2011.76	1984.75	35.7184	0.0029
27	2034.9	4.7046	0.002	2036.83	2013.68	30.7092	0.0076
28	2160.27	4.6015	0.0393	2183.42	2038.76	193.7647	0.4192
29	2279.86	4.362	0.0585	2299.15	2185.35	153.9843	0.2605
30	2312.65	4.3351	0.0292	2320.37	2301.08	26.2528	0.0603
31	2362.89	4.0407	0.0188	2385.95	2322.29	87.8763	0.2313
32	2395.59	3.9977	0.0672	2414.88	2385.95	40.3228	0.0686
33	2883.58	0.6239	0.0291	2887.44	2416.81	736.308	0.1424

SHIMADZU

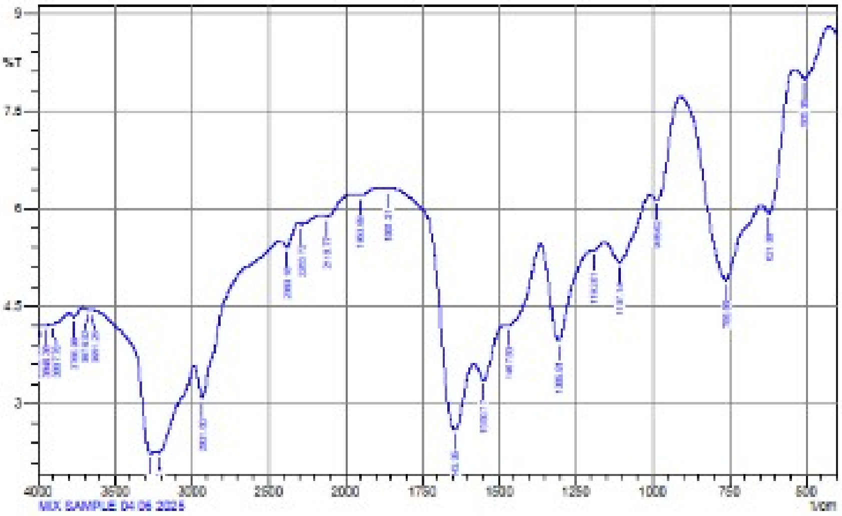

No.	Peak	Intensity	Corr. Intensity	Base (H)	Base (L)	Area	Corr. Area
1	505.35	7.9902	0.3326	536.21	428.2	116.5561	1.0455
2	621.08	5.9298	0.6025	646.15	536.14	126.1666	1.9328
3	759.95	4.8965	1.8705	906.54	648.08	316.5241	15.1595
4	985.62	6.1801	0.4136	1008.77	908.47	117.4185	1.3367
5	1107.14	5.1699	0.5546	1155.36	1010.7	181.6908	3.2298
6	1192.01	5.3552	0.0202	1197.79	1157.29	51.3027	0.0374
7	1305.81	3.9738	1.446	1363.67	1199.72	216.8367	9.0776
8	1487.83	4.2	0.1746	1483.26	1365.6	157.2549	2.1635
9	1550.77	3.3674	0.4281	1579.7	1485.19	135.0836	2.0242
10	1643.35	2.5953	1.6626	1842.02	1581.63	350.4329	12.3266
11	1861.31	6.3032	0.0036	1892.17	1851.66	46.6125	0.0068
12	1953.89	6.2026	0.0397	1977.04	1894.1	99.6967	0.134
13	2119.77	5.8824	0.0784	2156.42	1978.97	216.5555	0.4885
14	2283.72	5.786	0.0327	2304.94	2158.35	181.0428	0.1524
15	2380.16	5.4145	0.1903	2428.36	2306.86	162.6171	0.6002
16	2501.8	3.0901	0.6715	2963.88	2430.31	742.6614	5.7499
17	3217.27	2.2499	0.1324	3240.41	2966.81	395.3767	2.3257
18	3287.41	2.2434	0.1606	3626.17	3242.34	552.4478	0.9877
19	3651.25	4.436	0.008	3664.75	3643.53	26.6561	0.0102
20	3676.32	4.4436	0.0136	3705.26	3666.68	52.1061	0.0244
21	3756.98	4.3355	0.088	3797.84	3707.18	123.0006	0.3659
22	3907.78	4.2223	0.0107	3913.57	3809.41	142.4632	0.2136
23	3946.36	4.2082	0.0011	3948.29	3916.6	46.0868	0.0011
24	3992.65	4.1907	0.0039	4000.36	3948.29	71.7049	0.0096

Date/Time: 6/4/2025 2:55:35 AM
No. of Scans;
Resolution;
Apodization;

SHIMADZU

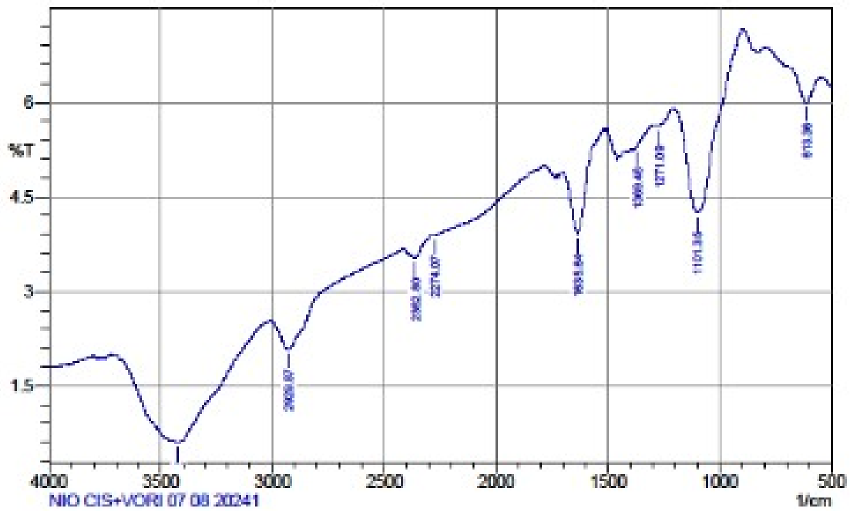

	Peak	Intensity	Corr. Intensity	Base (H)	Base (L)	Area	Corr. Area
1	613.36	5.5943	0.0136	616.29	563.57	74.5669	0.0568
2	1101.35	4.25	2.0607	1207.44	896.83	369.3741	23.0424
3	1271.09	5.6197	0.0643	1286.52	1209.37	95.8473	0.2518
4	1369.46	5.2693	0.0094	1371.39	1296.16	94.8056	0
5	1635.64	3.5026	1.2262	1699.29	1564.63	193.8613	7.5697
6	2274.07	3.8809	0.0094	2277.93	1915.31	499.3974	2.7027
7	2362.8	3.5213	0.1572	2389.8	2299.15	130.2752	0.8706
8	2929.87	2.0857	0.6007	3010.88	2414.88	911.0124	11.0625
9	3421.72	0.6036	1.5558	3680.18	3012.81	1261.4464	156.2466

Comment;
NIO CIS+VORI 07 08 20241

Date/Time; 8/7/2024 2:54:11 PM
No. of Scans;
Resolution;
Apodization;
User; Amman Uni

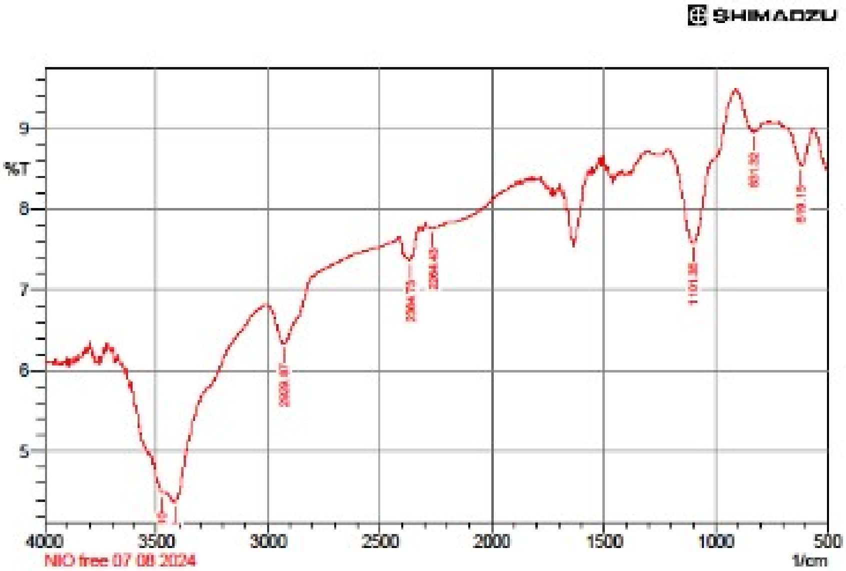

	Peak	Intensity	Corr. Intensity	Base (H)	Base (L)	Area	Corr. Area
1	619.15	8.5279	0.5011	716.59	561.29	162.613	1.5347
2	831.32	8.9536	0.3099	908.47	767.67	146.4729	1.0872
3	1101.35	7.5837	1.4283	1213.23	910.4	325.1355	9.9611
4	2264.43	7.7622	0.003	2266.36	1988.61	306.0808	0.7549
5	2364.73	7.37	0.2064	2389.8	2324.22	73.7474	0.4216
6	2929.87	6.3294	0.5922	3005.1	2416.81	673.854	3.8636
7	3419.79	4.3673	0.3196	3458.37	3007.02	559.4314	1.4254
8	3473.8	4.4823	0.0658	3531.66	3466.08	87.1534	0.2163

Comment;
NIO free 07 08 2024

Date/Time; 8/7/2024 2:42:54 PM
No. of Scans;
Resolution;
Apodization;
User; Amman Uni
